# Addition of Docetaxel to First-line Long-term Hormone Therapy in Prostate Cancer (STAMPEDE): Modelling to Estimate Long-term Survival, Quality-adjusted Survival, and Cost-effectiveness

**DOI:** 10.1016/j.euo.2018.06.004

**Published:** 2018-12

**Authors:** Beth S. Woods, Eleftherios Sideris, Matthew R. Sydes, Melissa R. Gannon, Mahesh K.B. Parmar, Mymoona Alzouebi, Gerhardt Attard, Alison J. Birtle, Susannah Brock, Richard Cathomas, Prabir R. Chakraborti, Audrey Cook, William R. Cross, David P. Dearnaley, Joanna Gale, Stephanie Gibbs, John D. Graham, Robert Hughes, Rob J. Jones, Robert Laing, Malcolm D. Mason, David Matheson, Duncan B. McLaren, Robin Millman, Joe M. O'Sullivan, Omi Parikh, Christopher C. Parker, Clive Peedell, Andrew Protheroe, Alastair W.S. Ritchie, Angus Robinson, J. Martin Russell, Matthew S. Simms, Narayanan N. Srihari, Rajaguru Srinivasan, John N. Staffurth, Santhanam Sundar, George N. Thalmann, Shaun Tolan, Anna T.H. Tran, David Tsang, John Wagstaff, Nicholas D. James, Mark J. Sculpher

**Affiliations:** a Centre for Health Economics, University of York, York, UK; b MRC Clinical Trials Unit at UCL, Institute of Clinical Trials and Methodology, University College London, London, UK; c Weston Park Hospital, Sheffield, UK; d The Institute of Cancer Research, London, UK; e The Royal Marsden NHS Foundation Trust, London, UK; f Rosemere Cancer Centre, Royal Preston Hospital, Preston, UK; g Dorset Cancer Centre, Poole Hospital NHS Foundation Trust, Poole, UK; h Swiss Group for Clinical Cancer Research (SAKK), Bern, Switzerland; i Kantonsspital Graubünden, Chur, Switzerland; j Royal Derby Hospital, Derby, UK; k Gloucestershire Oncology Centre, Cheltenham, UK; l Department of Urology, Leeds Teaching Hospitals NHS Trust, Leeds, UK; m Portsmouth Oncology Centre, Queen Alexandra Hospital, Portsmouth, UK; n Barking, Havering and Redbridge University Hospitals NHS Trust, Romford, UK; o Beacon Centre, Musgrove Park Hospital, Taunton, UK; p Mount Vernon Group, Mount Vernon Hospital, Northwood, UK; q University of Glasgow, UK; r St Luke's Cancer Centre, Royal Surrey NHS Trust, Guildford, UK; s School of Medicine, Cardiff University, Cardiff, UK; t University of Wolverhampton, Wolverhampton, UK; u Department of Oncology, Western General Hospital, Edinburgh, UK; v Centre for Cancer Research and Cell Biology, Queen's University, Belfast, UK; w Department of Oncology, East Lancashire Hospitals NHS Trust, Burnley, UK; x South Tees NHS Foundation Trust, Middlesbrough, UK; y Department of Oncology, Churchill Hospital, Oxford, UK; z Gloucestershire Royal Hospital Foundation Trust, Gloucester, UK; aa Sussex Cancer Centre, Royal Sussex County Hospital, Brighton, UK; bb Institute of Cancer Sciences, University of Glasgow, Glasgow, UK; cc Forth Valley Royal Hospital, Larbert, UK; dd Hull and East Yorkshire Hospitals NHS Trust, Hull, UK; ee Shrewsbury and Telford Hospitals NHS Trust, Shrewsbury, UK; ff Royal Devon & Exeter NHS Foundation Trust, Exeter, UK; gg Velindre Cancer Centre, Cardiff and School of Medicine, Cardiff University, Cardiff, UK; hh University of Nottingham, Nottingham, UK; ii Department of Urology, University Hospital Bern, Bern, Switzerland; jj Clatterbridge Cancer Centre, Birkenhead, UK; kk The Christie NHS Foundation Trust, Manchester, UK; ll Southend and Basildon Hospitals, Southend, UK; mm Swansea University College of Medicine, Swansea, UK; nn Queen Elizabeth Hospital, Edgbaston, Birmingham, UK

**Keywords:** Cost-effectiveness analysis, Docetaxel, Prostate cancer

## Abstract

**Background:**

Results from large randomised controlled trials have shown that adding docetaxel to the standard of care (SOC) for men initiating hormone therapy for prostate cancer (PC) prolongs survival for those with metastatic disease and prolongs failure-free survival for those without. To date there has been no formal assessment of whether funding docetaxel in this setting represents an appropriate use of UK National Health Service (NHS) resources.

**Objective:**

To assess whether administering docetaxel to men with PC starting long-term hormone therapy is cost-effective in a UK setting.

**Design, setting, and participants:**

We modelled health outcomes and costs in the UK NHS using data collected within the STAMPEDE trial, which enrolled men with high-risk, locally advanced metastatic or recurrent PC starting first-line hormone therapy.

**Intervention:**

SOC was hormone therapy for ≥2 yr and radiotherapy in some patients. Docetaxel (75 mg/m^2^) was administered alongside SOC for six three-weekly cycles.

**Outcome measurements and statistical analysis:**

The model generated lifetime predictions of costs, changes in survival duration, quality-adjusted life years (QALYs), and incremental cost-effectiveness ratios (ICERs).

**Results and limitations:**

The model predicted that docetaxel would extend survival (discounted quality-adjusted survival) by 0.89 yr (0.51) for metastatic PC and 0.78 yr (0.39) for nonmetastatic PC, and would be cost-effective in metastatic PC (ICER £5514/QALY vs SOC) and nonmetastatic PC (higher QALYs, lower costs vs SOC). Docetaxel remained cost-effective in nonmetastatic PC when the assumption of no survival advantage was modelled.

**Conclusions:**

Docetaxel is cost-effective among patients with nonmetastatic and metastatic PC in a UK setting. Clinicians should consider whether the evidence is now sufficiently compelling to support docetaxel use in patients with nonmetastatic PC, as the opportunity to offer docetaxel at hormone therapy initiation will be missed for some patients by the time more mature survival data are available.

**Patient summary:**

Starting docetaxel chemotherapy alongside hormone therapy represents a good use of UK National Health Service resources for patients with prostate cancer that is high risk or has spread to other parts of the body.

## Take Home Message

Docetaxel is a cost-effective intervention in patients with metastatic and nonmetastatic prostate cancer starting hormone therapy. Clinicians and National Health Service funding bodies should consider whether the evidence is now sufficiently compelling to support initiating docetaxel in patients with nonmetastatic disease.

## 1 Introduction

For many decades first-line treatment for locally advanced and metastatic prostate cancer (PC) has been based on long-term hormone therapy. The prognosis for these patients has improved in recent years with the licensing of agents that increase survival (docetaxel, abiraterone, enzalutamide, cabazitaxel, radium-223, and sipuleucel-T) and reduce morbidity (zoledronic acid and denosumab) [1]. These agents have all shown benefits in the setting of castrate-resistant PC (CRPC; ie, after first-line hormone therapy has ceased to work). More recently, the STAMPEDE trial is assessing various treatment approaches in the first-line, hormone naïve setting [2].

A number of randomised trials have been conducted to assess whether men with metastatic or high-risk localised PC starting hormone therapy would benefit from addition of docetaxel (with or without other agents) [3], [4], [5], [6]. Results from some of the largest of these trials, including STAMPEDE [7], have now emerged and been combined in a meta-analysis [8]. Collectively, these studies showed that six cycles of docetaxel extend survival and failure-free survival (FFS) for men with metastatic PC. For men with nonmetastatic PC, FFS was clearly improved by docetaxel; however, there were relatively few deaths, so statements about overall survival in this population remain uncertain. The National Health Service (NHS) in England currently funds docetaxel in newly-diagnosed men with metastatic PC who are starting hormone therapy or who have started hormone therapy within the last 12 wk. There is currently no NHS policy statement regarding the use of docetaxel among patients with high-risk nonmetastatic PC commencing hormone therapy.

In this cost-effectiveness study, we use data from the “docetaxel comparison” of the STAMPEDE trial and modelling methods to assess whether (1) the cost-effectiveness evidence supports the decision made by NHS England to fund docetaxel for patients with metastatic PC; and (2) whether this recommendation should be extended to individuals with nonmetastatic PC for whom funding is not currently mandated in the UK.

## 2 Patients and methods

### 2.1 Overview

The methods for this economic evaluation follow the reference case set out by the National Institute for Health and Care Excellence (NICE) [8] and the reporting adheres to the Consolidated Health Economic Evaluation Reporting Standards statement [9]. We used a modelling approach to predict the lifetime experience of patients receiving each intervention. In line with the NICE reference case, the model uses a lifetime time horizon, health outcomes are quantified in terms as quality-adjusted life years (QALYs), which provide a means of reflecting patient morbidity and mortality, and a 3.5% annual discount rate (the rate at which costs and outcomes incurred in the future are converted to their value today) is applied for costs and outcomes.

A modelling rather than within-trial analysis is necessary as approximately half of the patients in these STAMPEDE research comparisons were still alive at the time data were frozen for the primary survival analysis. It was therefore necessary to account for the remainder of their projected life experience using a predictive model (hence we use the term *predicted survival* in our results). The perspective for this analysis is the UK NHS and Personal and Social Services. Results are presented in terms of the incremental cost-effectiveness ratio (ICER) for SOC plus docetaxel, that is, its additional cost per QALY gained compared to SOC alone. If docetaxel reduces costs and increases predicted QALYs, it is termed *dominant*.

### 2.2 STAMPEDE

We used the STAMPEDE trial as the main source of data to assess the cost-effectiveness of adding docetaxel to SOC as STAMPEDE represents the largest trial of docetaxel in this setting, is reflective of UK practice, and collected extensive data on patient health-related quality of life (HRQoL) and resource use. Full details of the STAMPEDE trial can be found elsewhere [2], [7], [10], [11]. In brief, the trial uses a multiarm, multistage (MAMS) platform design to test whether addition of treatments at the time of long-term hormone therapy initiation improves overall survival (OS). STAMPEDE recruits men with high-risk, locally advanced, metastatic or recurrent PC who are starting first-line long-term hormone therapy, and has enrolled more than 9000 men to ten different comparisons so far [7], [12]. The first set of comparisons from STAMPEDE revealed that docetaxel chemotherapy improved survival and failure-free survival (FFS) but was accompanied by an increase in adverse events. SOC-only comprised hormone therapy for at least 2 yr and radiotherapy was encouraged for men with node-negative nonmetastatic PC until November 2011, when it was mandated, and was optional throughout in those with node-positive nonmetastatic disease. Docetaxel (75 mg/m^2^) [12] was administered alongside SOC (SOC + Doc) in six three-weekly cycles with prednisolone 10 mg daily.

### 2.3 Estimation of disease progression

**Fig. 1:**
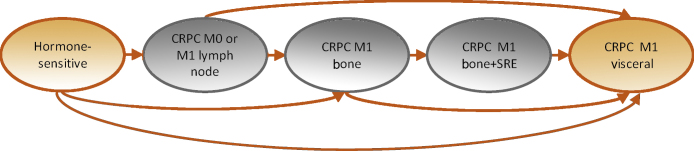
Model structure. Patients start treatment in the hormone-sensitive health state and then progress to the castrate-resistant prostate cancer (CRPC) states. At treatment failure, patients enter the CRPC state that reflects their worst previous disease event (with the worst event being visceral metastases, then bone metastases with history of a skeletal-related event [SRE], then bone metastases without an SRE, then CRPC with no metastases or only lymph-node metastases). Further events can cause movement to more severe health states. Death due to prostate cancer or non–prostate cancer is possible from any of the health states (not shown for parsimony).

The model structure was developed to reflect the natural history of PC patients on the basis of a review of observational data, clinical guidelines, and clinical advice (Fig. 1). A patient-level simulation approach was used to generate lifetime predictions for the cohort of patients enrolled in STAMPEDE [13]. This approach provides a simple way of reflecting time-varying rates of clinical events. Predictions were generated as if all patients enrolled in the original comparisons in STAMPEDE were allocated to SOC, then as if all patients were allocated to SOC + Doc to eliminate chance imbalances in patient characteristics between comparators.

A multistate survival-analytic approach was used to estimate the rate at which individuals move through the health states in STAMPEDE [14], [15], [16]. Parametric survival models were fitted to allow extrapolation of the estimated hazard rates beyond the data collected in the trial period for those still alive (censored) at the preplanned analysis [17]. The first transition represents time to treatment failure and was estimated as a function of treatment allocation and baseline patient characteristics that have previously been found to be prognostic [10], [11]. Transitions beyond the point of treatment failure were estimated conditional on patients’ treatment allocation and time of failure. Robust data on outcomes beyond the onset of metastatic CRPC were not available from STAMPEDE for patients with nonmetastatic PC at baseline according to the follow-up duration currently available. Data for metastatic CRPC cases who had metastatic disease at baseline were therefore assumed to apply to metastatic CRPC cases with nonmetastatic disease at baseline. This assumption was supported by the literature [18] and clinical opinion.

### 2.4 HRQoL and costs

HRQoL was reflected in the model as a function of patients’ baseline characteristics, the health states they occupied over time, and the toxicity effects of docetaxel. HRQoL was estimated using the EQ-5D three-level version (EQ-5D-3L; https://euroqol.org/eq-5d-instruments/eq-5d-3l-about/) collected throughout follow-up for the first 700 patients randomised to STAMPEDE. Patient responses to the EQ-5D-3L questionnaire were converted to HRQoL weights using UK general population preference data [19]. The resulting EQ-5D scores for all time points were subjected to regression analysis to predict EQ-5D scores conditional on patients’ characteristics at baseline considered to be predictive of HRQoL according to clinical opinion (age, World Health Organisation [WHO] status, nodal stage), chemotherapy impact, and health state, as shown in Fig. 1. It was assumed that docetaxel toxicity impacted on HRQoL for 1 yr. This was based on data from STAMPEDE that indicated that the proportion of patients reporting worst adverse event ever as grade 3 or higher was initially higher in the SOC + Doc group, but that this difference no longer existed at approximately 1 yr [7]. The resulting HRQoL weights were allocated according to patient baseline characteristics, treatment allocation, and health state to generate estimates of lifetime QALYs.

Costs were reflected in the model for docetaxel acquisition and administration; adverse events; disease and toxicity monitoring; general disease management (including hormone therapy; concomitant and postprogression drugs, radiotherapy, and procedures; and unscheduled primary and secondary care for PC-related hospital attendance); acquisition and administration of life-extending therapies (docetaxel, abiraterone, enzalutamide, cabazitaxel, and radium-223); and end-of-life care (Table 1). Resource use data were taken from STAMPEDE where available, supplemented with data from the literature and clinical opinion.

**Table 1:** Cost data used in analysis

Cost category	Cost used, £ (95% CI)	Source
Docetaxel acquisition, administration and monitoring costs (per course) WHO status 0 and age <60 yr WHO status 0 and age 60–64 yr WHO status 0 and age 65–69 yr WHO status 0 and age ≥70 yr WHO status 1–2 and age <60 yr WHO status 1–2 and age 60–64 yr WHO status 1–2 and age 65–69 yr WHO status 1–2 and age ≥70 yr	1897 (1777–1999) 1947 (1844–2025) 1847 (1733–1945) 1610 (1468–1738) 1422 (1044–1772) 1524 (1236–1822) 1798 (1561–2007) 1663 (1413–1873)	Analysis of STAMPEDE individual patient data (Supplementary material)
Adverse event costs (per event) Additional cost associated with neutropenia Additional cost associated with febrile neutropenia	128 (NA^a^) 1363 (NA^a^)	NHS reference costs [23]
Annual cost of monitoring Hormone-sensitive year 1 Hormone-sensitive years 2–5 Castrate-resistant On chemotherapy, abiraterone, or enzalutamide	684 (NA^b^) 538 (NA^b^) 1764 (NA^b^) 2256 (NA^b^)	Previous studies [31], NICE appraisals [32], expert opinion, NHS reference costs [23]
Annual costs of long-term management Constant c First year on SOC First year on SOC + Doc Age 60–64 yr Age 65–69 yr Age ≥70 yr WHO status 1 and 2 Nodal status N+ Nodal status NX (unknown) Health state: hormone-sensitive M1 bone Health state: hormone-sensitive M1 visceral Health state: CRPC M0 or M1 lymph node Health state: CRPC M1 bone Health state: CRPC M1 bone + SRE Health state: CRPC M1 visceral	1209 (970–1453) 222 (107–342) 762 (524–995) −222 (−479 to 38) −177 (−429 to 74) 42 (−289 to 369) 390 (110–663) 279 (92–456) 474 (−337 to 1278) 876 (642–1123) 342 (51–661) 633 (448–799) 2295 (1978–2617) 3507 (2890–4075) 2397 (1723–3088)	Analysis of STAMPEDE individual patient data (Supplementary material)
Annual life-extending therapy costs Health state: hormone-sensitive M0 or M1 lymph node Year 1 Year 2 Year 3 Health state: hormone-sensitive M1 bone Year 1 Year 2 Year 3 Health state: hormone-sensitive M1 visceral Year 1 Year 2 Year 3 Health state: CRPC M0 or M1 lymph node Year 1 Year 2 Year 3 Health state: CRPC M1 bone Year 1 Year 2 Year 3 Health state: CRPC M1 bone + SRE Year 1 Year 2 Year 3 Health state: CRPC M1 visceral Year 1 Year 2 Year 3	204 (7–615) 315 (7–856) 162 (55–286) 1278 (475–2141) 2469 (960–4234) 465 (213–1044) 9 (8–10) 9 (7–9) 96 (8–1567) 9831 (4894–16 010) 5226 (1020–9814) 3534 (1907–5708) 14 661 (10 914–18 671) 7488 (3875–11 599) 7344 (3758–8576) 12 861 (7774–18 112) 9120 (3888–14 899) 11 541 (5002–15 186) 7977 (1255–16 229) 24 534 (7–123,060) 3822 (7–9557)	Analysis of STAMPEDE individual patient data
End of life (per prostate cancer-related death)	6687 (535–20 257)	Round et al [33]

CI = confidence interval; WHO = World Health Organisation; CRPC = castrate-resistant prostate cancer; SRE = skeletal-related event; NHS = National Health Service; NICE = National Institute for Health and Care Excellence.

a Confidence interval not available as data represents a unit cost.

b No confidence interval available as data obtained from expert opinion and NICE guidance.

c The impact of each covariate is shown relative to a reference patient with M0 hormone-sensitive disease, not on the first year of treatment, aged <60 yr, with WHO status 0, and node-negative disease.

General disease management costs were analysed using a regression approach that estimated costs conditional on patient baseline characteristics considered to be predictive of costs (age, WHO status, nodal stage), whether or not the individual was within 1 yr of receiving docetaxel, and their health state (as shown in Fig. 1). Life-extending therapy costs were estimated for each health state and for each study arm, as choice of life-extending therapy was found to differ substantively between arms [7]. Monitoring costs were assumed to differ across health states, and whether or not patients were in receipt of active therapy requiring more intensive monitoring. Unit costs were obtained from standard UK sources [20], [21], [22], [23]. Generic drug costs were taken from the electronic market information tool (eMit) [21] where possible, as these reflect actual prices paid by NHS hospitals for docetaxel and other relevant products.

An androgen-receptor pathway inhibitor (abiraterone or enzalutamide) is currently the first-line treatment choice for the majority of patients with metastatic CRPC who receive a life-extending therapy [24]. In STAMPEDE, these AR-pathway inhibitor treatments were used more frequently after the onset of metastatic CRPC if patients were allocated to SOC + Doc in the hormone-naïve setting, as part of the trial, than if they were allocated to SOC [7]. This reflects the early licensing and reimbursement approvals of the AR pathway inhibitors. NICE approved their use in patients who had received prior chemotherapy in 2012, and this was extended to all patients at first relapse in 2016 (although earlier in England via the Cancer Drugs Fund). To reflect current practice, we therefore applied the life-extending therapy usage observed in the SOC + Doc arm to the SOC arm and used data from the COU-AA-302 trial [25] to model the better outcomes expected for patients allocated to SOC. Given the limited data from STAMPEDE on life-extending therapy use in the longer term, data were pooled across study arms and time periods from the third year onwards for each health state.

### 2.5 Sensitivity analysis

Sensitivity analyses were conducted for the rates of progression through the clinical health states, the nature of current treatment practice, and the costs of branded and generic drugs. A probabilistic sensitivity analysis was also conducted to jointly reflect all parameter uncertainty. Results are presented separately for nonmetastatic and metastatic cases given the differences in prognosis and long-term care.

As there is uncertainty about OS results for patients with nonmetastatic PC given the immature data from STAMPEDE, a sensitivity analysis was undertaken to explore the impact of inferring an OS benefit using predictive modelling from patients who had metastatic PC at baseline. This was simulated by assigning a much higher rate of metastases incidence to patients with nonmetastatic CRPC in the SOC + Doc arm. Under this scenario, gains in the SOC + Doc arm in terms of time spent failure-free are offset by losses in time spent with CRPC.

The small group of patients with nonregional lymph node metastases as their only site(s) of metastases (M1a disease) are grouped here with the patients with nonmetastatic PC. This is because although their prognosis is poorer than for those with nonmetastatic PC, their outcomes are closer to those for patients with advanced localised PC than to those for individuals whose disease involved additional distant sites [10], [11]. Results were also examined by predicted time to failure to identify any variation in cost-effectiveness according to patient prognosis. Further details regarding the study methods and data are provided in the Supplementary material.

## 3 Results

### 3.1 Patient prognosis over time: nonmetastatic PC

**Fig. 2:**
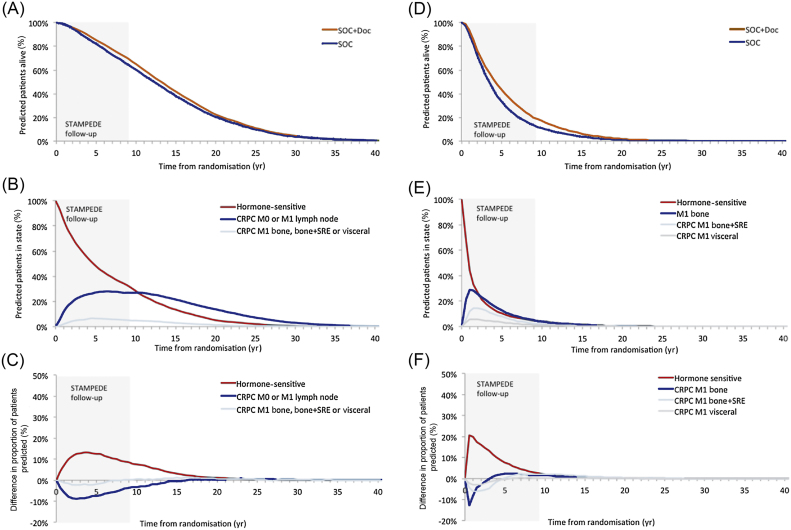
Predicted patient prognosis over time. (A) Overall survival for patients with M0 disease. (B) Proportion of patients with M0 disease receiving standard of care (SOC) by health state. (C) Difference in proportion of patients with M0 disease in each health state (SOC + docetaxel (Doc) minus SOC). (D) Overall survival for patients with M1 disease. (E) Proportion of patients with M1 disease receiving SOC by health state. (F) Difference in proportion of patients with M1 disease in each health state (SOC + Doc minus SOC). CRPC = castrate-resistant prostate cancer; SRE = skeletal-related event, M0 = nonmetastatic, M1 = metastatic. The grey shaded area denotes the duration of patient follow-up in STAMPEDE.

The model predicted that a higher proportion of nonmetastatic cases who were allocated to SOC + Doc would be alive at each time point compared to SOC (Fig. 2A). These patients spend their time predominantly without bone, bone + skeletal-related event (SRE), or visceral metastases with or without treatment failure (Fig. 2B). A higher proportion of patients in the SOC + Doc arm than in the SOC arm were predicted to be failure-free, and a lower proportion were alive with treatment failure (Fig. 2C). The model predicted that docetaxel extended unrestricted mean survival duration by 0.78 yr (SOC 13.33 yr, SOC + Doc 14.11 yr); extended predicted, unrestricted mean time in the failure-free (hormone-sensitive) state by 1.42 yr (SOC 7.08 yr, SOC + Doc 8.50 yr); and reduced predicted, unrestricted mean time in the CRPC states by 0.61 yr (SOC 5.33 yr, SOC + Doc 4.72 yr) for the CRPC M0/M1 lymph node state and 0.03 yr (SOC 0.92 yr, SOC + Doc 0.89 yr) for the CRPC M1 bone, bone + SRE, or visceral states.

### 3.2 Patient prognosis over time: metastatic PC

A higher proportion of patients with metastatic PC who received SOC + Doc were predicted to be alive at each time point compared to those receiving SOC (Fig. 2D). These patients were projected to spend their time predominantly with metastatic disease without treatment failure or with treatment failure and bone metastases (Fig. 2E). A higher proportion of patients in the SOC + Doc arm than in the SOC arm were failure-free, and a lower proportion were alive with treatment failure (Fig. 2F). Docetaxel extended predicted, unrestricted mean survival duration by 0.89 yr (SOC 4.90 yr, SOC + Doc 5.79 yr); extended predicted, unrestricted mean time in the failure-free state (hormone-sensitive) by 0.99 yr (SOC 2.04 yr, SOC + Doc 3.03 yr); and reduced predicted, unrestricted mean time in the CRPC M1 states by 0.10 yr (SOC 2.86 yr, SOC + Doc 2.76 yr).

### 3.3 HRQoL and costs

**Fig. 3:**
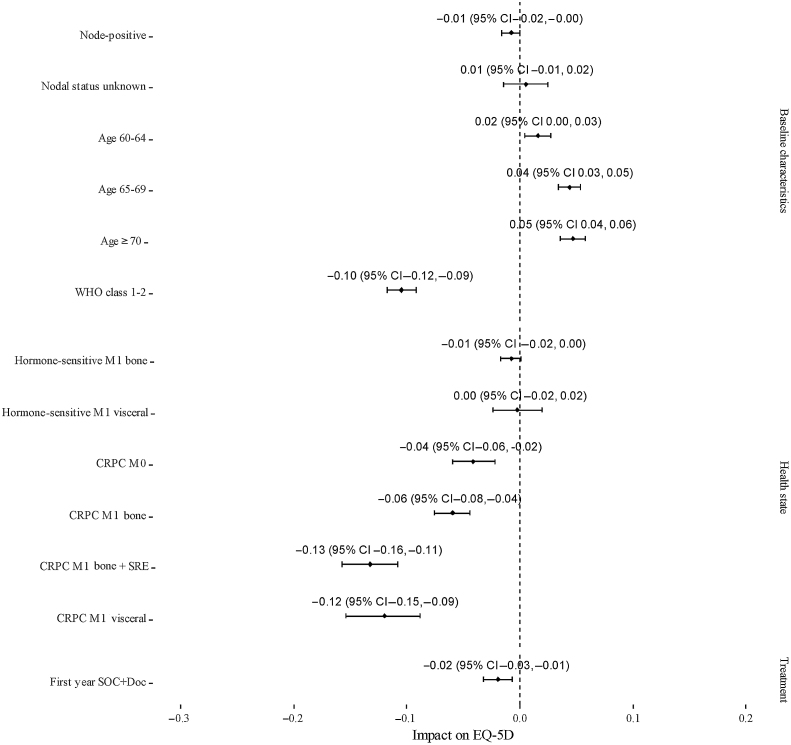
Impact of baseline characteristics, health state, and treatment allocation on patient health-related quality of life (HRQOL) in STAMPEDE. This graph presents the results of an analysis of EQ-5D data obtained from STAMPEDE adjusted for baseline characteristics, treatment allocation, and current health state. Data were collected at baseline and at follow-up visits: every 6 wk for the first 6 mo, then every 12 wk up to 2 yr, every 6 mo up to 5 yr, and annually thereafter. The impact of each covariate on HRQOL is shown relative to a reference patient with nonmetastatic disease, World Health Organisation class 0, age ≤60 yr, and node-negative in their first year of standard of care. Positive values indicate better and negative values indicate worse HRQOL relative to the reference patient. CRPC = castrate-resistant prostate cancer; SRE = skeletal-related event, M0 = nonmetastatic, M1 = metastatic.

In the first year following randomisation, patients who received docetaxel experienced a small decrement in HRQoL (Fig. 3). Patients with CRPC have impaired HRQoL, particularly those who have bone metastases and have experienced an SRE, and those who have visceral disease (Fig. 3). The cost data used in the model are shown in Table 2. The monitoring, management, and life-extending therapy costs are much higher for patients with CRPC in the model.

### 3.4 Cost-effectiveness results: nonmetastatic PC

For patients with nonmetastatic PC, the higher costs associated with acquiring and administering docetaxel, managing adverse events, and managing patients over their predicted longer life expectancy were offset by lower monitoring costs and life-extending therapy costs, as patients would experience a shorter period of their lives with CRPC (Table 2). The net impact of these effects is that docetaxel is predicted to save the NHS £251 per treated patient over patients’ lifespan. The predicted improvement in QALYs associated with SOC + Doc was 0.39 per patient, with patients receiving SOC + Doc accruing additional QALYs in the failure-free state and fewer QALYs in the CRPC states. The addition of docetaxel to SOC therefore offers health benefits and cost savings to the NHS (ie, it is a dominant treatment).

**Table 2:** Cost-effectiveness results

	Nonmetastatic prostate cancer			Metastatic prostate cancer		
	SOC	SOC + Doc	Difference	SOC	SOC + Doc	Difference
Costs (UK pounds, discounted)						
Docetaxel	–	1791	1791	–	1761	1761
Monitoring	10 912	10 451	−461	5471	5641	170
Management including toxicities	17 400	18 574	1174	14 415	16 555	2139
Life-extending therapies	24 679	21 964	−2715	27 716	26 611	−1105
End-of-life care	2124	2084	−40	4864	4687	−177
Total	55 114	54 863	−251	52 466	55 253	2787
Life years (undiscounted)	13.33	14.11	0.78	4.90	5.79	0.89
QALYs (discounted)						
Failure-free	4.44	5.27	0.83	1.40	2.02	0.63
Post-failure	3.04	2.60	−0.44	1.61	1.49	−0.12
Total	7.48	7.87	0.39	3.01	3.51	0.51
ICER (UK pounds/QALY)			Dominant			5,514

SOC = standard of care; Doc = docetaxel; ICER = incremental cost-effectiveness ratio; QALY = quality-adjusted life year.

### 3.5 Cost-effectiveness results: metastatic PC

For patients with metastatic PC, the incremental costs associated with acquiring and administering docetaxel, managing adverse events, and managing patients over a longer life expectancy were only partially offset by savings on life-extending therapy costs, resulting in an incremental cost for SOC + Doc of £2787 per patient. This is because increasing the life expectancy of patients with metastatic PC is more costly, and because these patients experience a more similar period with CRPC regardless of the original treatment allocation. The predicted discounted improvement in QALYs associated with SOC + Doc was 0.51 per patient, with patients receiving SOC + Doc accruing additional QALYs in the failure-free state and slightly fewer QALYs in the CRPC states. The addition of docetaxel to SOC is therefore associated with an ICER of £5514/QALY, which is considerably lower than cost-effectiveness thresholds currently used in the UK NHS (which range from £13 000 to £30 000/QALY [26], [27]).

### 3.6 Sensitivity analyses

The results were similar across risk quartiles defined according to predicted time to progression for both nonmetastatic and metastatic groups. The probabilistic sensitivity analysis indicated a very high probability (>99%) that docetaxel is cost-effective in both nonmetastatic and metastatic PC using the base-case model specifications. Two sensitivity analyses increased the ICER above £13 000/QALY. First, when the British National Formulary price for docetaxel was used (which is considerably higher than the current price the NHS pays) [20], [21], the ICER for docetaxel increased to £10 610/QALY for nonmetastatic and £13 868/QALY for metastatic cases. When patients in the SOC arm were assumed to be less likely to receive abiraterone or enzalutamide in CRPC (as observed in STAMPEDE), the ICER increased to £13 299/QALY for nonmetastatic and £18 342/QALY for metastatic cases. Inclusion of data from a meta-analysis of all relevant trials in this area resulted in ICERs of approximately £8000/QALY for both nonmetastatic and metastatic cases.

The sensitivity analysis to explore the impact of assuming no survival gain in nonmetastatic PC showed that the QALY gain was much smaller than the base-case assumption, which predicted an OS benefit from patients who had metastatic PC at baseline. This is because this alternative assumption reflects only gains to patient HRQoL (0.04 QALYs). The cost saving observed is higher (£3128) than in the base case, as patients in the SOC + Doc arm spend less time accruing the management, monitoring, and life-extending therapy costs associated with CRPC. Docetaxel therefore remains dominant in this scenario.

## 4 Discussion

We found that addition of docetaxel to SOC represents a cost-effective use of UK NHS resources in both metastatic and nonmetastatic PC. Further work will be reported on the cost-effectiveness of abiraterone plus prednisolone for men starting long-term androgen deprivation therapy following positive survival results from STAMPEDE [28].

Following reports from trials evaluating the benefit of docetaxel addition for men with metastatic or high-risk localised PC [3], [4], [7], [8], NHS England commissioned NICE to review the evidence for docetaxel for patients with metastatic PC starting long-term hormone therapy [29], [30]. On the basis of this review, NHS England concluded that there was sufficient evidence to support routine funding of docetaxel for men newly diagnosed with metastatic PC who were starting hormone therapy or had started hormone therapy within the last 12 wk. No evidence review or NHS policy statement has yet been made in relation to the use of docetaxel for patients with high-risk nonmetastatic PC commencing hormone therapy. These economic analysis results suggest that this position should be reconsidered.

The implications for patients with nonmetastatic PC deserve further discussion. Given the cytotoxic nature of docetaxel treatment, many clinicians will require a clinically relevant and statistically significant improvement in OS to support its use. Unsurprisingly, at the first report of survival data from STAMPEDE, there was not a statistically significant improvement in OS for patients with nonmetastatic PC (hazard ratio [HR] 0.95, 95% confidence interval [CI] 0.62–1.47), or evidence of heterogeneity of the treatment effect compared to metastatic PC; a meta-analysis of all randomised controlled trial data in non-metastatic patients showed a trend towards improvement in OS that does not reach statistical significance (HR 0.87, 95% CI 0.69–1.09) [8].

Our model did not use these data as the sole basis for informing estimates of survival duration for patients with nonmetastatic PC, as the data were considered too immature to provide robust long-term predictions. Instead we predicted OS by estimating the impact of treatment failure on rates of subsequent metastases and mortality. This approach required extrapolation of information on outcomes in M1 CRPC from those who had metastases at baseline to those who did not, which introduces an additional level of uncertainty to the results, and clinicians may be reluctant to accept a survival advantage for patients with nonmetastatic PC until mature trial data demonstrate this. Nonetheless, even if it is assumed that SOC + Doc does not improve OS, our model predicts that it will result in an increase in QALYs compared to SOC. This is because the short-term negative effects of chemotherapy are offset by the HRQoL benefits of delaying the onset of CRPC. Furthermore, the cost saving on adding docetaxel is even higher under this assumption because SOC + Doc incurs lower costs as a result of less time spent in the CRPC state. Hence, docetaxel remains dominant in this scenario.

The modelling was associated with other uncertainties that are inevitable when attempting to infer the lifetime consequences of a treatment decision from trial data, and when attempting to predict the impact of changes to treatment practice. These included the fact that because of regulatory developments over the course of the trial, AR pathway inhibitors were more frequently used after the onset of metastatic CRPC in the hormone-naïve setting in STAMPEDE patients allocated to SOC + Doc rather than to SOC. The model was adjusted, using best available evidence [24], to reflect the fact that SOC patients would now be expected to access these treatments to the same degree. Sensitivity analysis showed that cost-effectiveness was somewhat dependent on this assumption, but more so in patients with metastatic PC, as many patients with nonmetastatic PC will die without developing M1 CRPC. Nonetheless, our work suggests that docetaxel remains cost-effective as long as its acquisition cost remains low, and the AR pathway inhibitors represent the mainstay of treatment in metastatic CRPC regardless of earlier treatment choices.

The analysis adheres to the methods used for NICE technology appraisals which are influential internationally. Clinical, HRQoL, and resource data from STAMPEDE, and hence the results of the cost-effectiveness analysis, are relevant to the NHS in England. The cost of specific resources consumed by patients may be different in other countries, including the acquisition cost of docetaxel if NHS hospitals are able to achieve lower prices than their counterparts elsewhere. However, clinical practice for these patients is similar internationally [24], suggesting that the magnitude of benefits estimated in the analysis is likely to be generalisable to other settings. Under most scenarios considered here, we found that adding docetaxel was cost-effective. Whether this is the case elsewhere will depend on unit costs, including drug prices, and the cost-effectiveness thresholds of different systems. The authors are willing to collaborate in adapting the model for other jurisdictions.

## 5 Conclusions

Docetaxel is a cost-effective intervention in patients with metastatic PC. For patients with nonmetastatic PC, our analysis suggests that treatment is also very likely to be cost-effective, and this is the case whether or not a survival advantage is included. Clinicians should consider carefully whether the evidence is now sufficiently compelling to support initiating docetaxel in selected patients with high-risk nonmetastatic PC currently starting hormone therapy.

Data are available for bona fide researchers on request from the authors.

  ***Author contributions***: Beth S. Woods had full access to all the data in the study and takes responsibility for the integrity of the data and the accuracy of the data analysis.  

*Study concept and design*: Woods, Sideris, Sydes, Spears, Parmar, James, Sculpher.

*Acquisition of data*: All authors.

*Analysis and interpretation of data*: All authors.

*Drafting of the manuscript*: Woods.

*Critical revision of the manuscript for important intellectual content*: All authors.

*Statistical analysis*: Woods, Sideris.

*Obtaining funding*: Sculpher, Sydes, James.

*Administrative, technical, or material support*: None.

*Supervision*: Sculpher, Woods.

*Other*: None.  

***Financial disclosures:*** Beth S. Woods certifies that all conflicts of interest, including specific financial interests and relationships and affiliations relevant to the subject matter or materials discussed in the manuscript (eg, employment/affiliation, grants or funding, consultancies, honoraria, stock ownership or options, expert testimony, royalties, or patents filed, received, or pending), are the following: Beth S. Woods has received payment for consultancy services from Servier Laboratories Ltd. Eleftherios Sideris reports no conflicts of interest at the time the research was conducted but is now an employee of Roche Products Ltd. Matthew R. Sydes reports personal payments and travel expenses from Eli Lilly, personal payments from Sanofi-Genzyme, unrestricted educational grants to his institution from Clovis Oncology, unrestricted educational grants plus drug and distribution costs to his institution from Astellas, Janssen, Novartis, and Pfizer, and unrestricted educational grants plus discounted drug to his institution from Sanofi-Genzyme. Melissa R. Gannon reports educational grants to her institution from Sanofi Aventis, Pfizer, Novartis, Janssen, and Astellas. Mahesh K.B. Parmar reports unrestricted educational grants plus drug and distribution costs to his institution from Astellas, Janssen, Novartis, and Pfizer, unrestricted educational grants to his institution from Clovis Oncology, and unrestricted educational grants plus discounted drug to his institution from Sanofi-Genzyme. Gerhardt Attard reports speaker fees from Sanofi Aventis. Alison J. Birtle reports receiving honoraria from Janssen, Astellas, Sanofi, Bayer, and AstraZeneca. Richard Cathomas reports receiving honoraria from Sanofi, Janssen, Astellas, and Bayer. David P. Dearnaley reports honararia and consultancy fees from Takeda, Amgen, Astellas, Sandoz, and Janssen Pharma, research funding from Cancer Research UK, and a financial interest in abiraterone via the Institute of Cancer Research Rewards to Inventors programme. Malcolm D. Mason reports honoraria from Sanofi Aventis and Janssen. Joe M. O'Sullivan reports honoraria from Sanofi, Janssen, and Bayer. Christopher C. Parker reports honoraria from AAA, Bayer, and Janssen. Narayanan N. Srihari reports travels grants from Janssen, Pfizer, and Aventis. Shaun Tolan reports travel bursaries from Astellas, Janssen, and Sanofi and honoraria from Astellas. John Wagstaff reports consultancy fees from Astellas and Janssen. Nicholas D. James reports receiving grant support, drug supplies and distribution, lecture fees, and advisory board fees from Janssen, Astellas Pharma, and Novartis, grant support, drug supplies and distribution, and lecture fees from Janssen and Pfizer, grant support and drug supplies and distribution from Janssen and Clovis Oncology, and grant support, discounted drug supplies, lecture fees, advisory board fees, and travel assistance from Sanofi-Aventis. Mark J. Sculpher reports consultancy for a number of pharmaceutical companies but none relating to the products considered in this paper. The remaining authors have nothing to disclose.  

***Funding/Support and role of the sponsor*****:** This study was supported by the UK Medical Research Council (delegation to Swiss Group for Cancer Clinical Research [SAKK] in Switzerland) grant number MRC_MC_UU_12023/25 and the following funders: Cancer Research UK (grant number CRUK_A12459), Medical Research Council, Astellas, Clovis Oncology, Janssen, Novartis, Pfizer, and Sanofi-Aventis. The sponsors played no direct role in the study.
