# Supplementary material for: Addition of Docetaxel to First-line Long-term Hormone Therapy in Prostate Cancer (STAMPEDE): Modelling to Estimate Long-term Survival, Quality-adjusted Survival, and Cost-effectiveness
Source: Eur Urol Oncol. 2018 Dec;1(6):449–58. doi: 10.1016/j.euo.2018.06.004 (PMC6692495; doi:10.1016/j.euo.2018.06.004)
Supplement: Supplementary file 1 [file mmc1.docx]

**Supplementary material**

[Estimation of progression through disease states 3](#_Toc500782192)

[Patient heterogeneity 4](#_Toc500782193)

[Multi-state modelling 5](#_Toc500782194)

[Survival analysis 6](#_Toc500782195)

[Time to first event 6](#_Toc500782196)

[Time to subsequent events 9](#_Toc500782197)

[Time to non-prostate cancer mortality 12](#_Toc500782198)

[Assessing the validity of model predictions 14](#_Toc500782199)

[Quality of life weights 17](#_Toc500782200)

[Data collection 17](#_Toc500782201)

[Statistical analysis 17](#_Toc500782202)

[Resource use and costing data 19](#_Toc500782203)

[Data collection 19](#_Toc500782204)

[Hormone therapy 20](#_Toc500782205)

[Trial treatment 22](#_Toc500782206)

[Concomitant treatments 23](#_Toc500782207)

[Post-progression therapies 23](#_Toc500782208)

[Hospital and primary care service use 27](#_Toc500782209)

[Adverse events 28](#_Toc500782210)

[Patient monitoring 28](#_Toc500782211)

[End of life care 31](#_Toc500782212)

[Statistical analysis 31](#_Toc500782213)

[Reflecting current treatment practice in the UK 33](#_Toc500782214)

[Evaluation of the disease model 33](#_Toc500782215)

[Sensitivity analyses conducted 34](#_Toc500782216)

[STAMPEDE investigators and trial committees 39](#_Toc500782217)

[References 43](#_Toc500782218)

## Estimation of progression through disease states

The matrix of all possible transitions in the model is presented in Table 1, and the number of transitions made by patients within STAMPEDE as Table 2. Patients entered the model in the non-progressed (castration-naïve) state. Patients at trial entry could be either non-metastatic or have lymph node metastases (M0/M1 lymph node) or have bone or visceral metastases (M1 bone/visceral). Patients classed as having liver, lung or “other” metastases were considered to have visceral metastases at baseline. The castrate resistant prostate cancer (CRPC) states represent treatment failure. Severity in CRPC is categorised from least to most severe (i.e. M0 or M1 lymph node, M1 bone, M1 bone+skeletal related event (SRE), M1 visceral).

Possible patient transitions in the model depend on whether patients are M0/M1 lymph node, M1 bone, or M1 visceral at baseline. For M0/M1 lymph node patients the model allows patients to fail treatment without bone or visceral metastases or with each type of metastases; then patients can progress to more severe health states. The structure for patients who are M1 bone/visceral at trial entry is very similar. The only difference is that M1 patients cannot occupy the treatment failure without bone or visceral metastases health state (CRPC M0 or M1 lymph node). Patients with CRPC may have multiple types of metastases and are classified according to the most severe type of metastases they have experienced, either at baseline or during the trial. Health states do not attempt to capture other baseline prognostic factors; these are captured separately by including baseline prognostic factors in the regression models that inform the model. Death can occur from any health state in the model. Prostate cancer and non-prostate cancer mortality are modelled as separate processes.

Supplementary Table 1: Matrix of all possible transitions in model (numbered)

| To  From | CRPC M0 / M1 lymph | CRPC M1 Bone | CRPC M1 Bone+SRE | CRPC M1 Visceral | PC death | Non-PC death |
| --- | --- | --- | --- | --- | --- | --- |
| M0 / M1 lymph | (1) | (2) |  | (3) | (4) | (5) |
| M1 bone |  | (6) |  | (7) | (8) | (9) |
| M1 visceral |  |  |  | (10) | (11) | (12) |
| CRPC M0 / M1 lymph |  | (13) |  | (14) | (15) | (16) |
| CRPC M1 Bone |  |  | (17) | (18) | (19) | (20) |
| CRPC M1 Bone+SRE |  |  |  | (21) | (22) | (23) |
| CRPC M1 Visceral |  |  |  |  | (24) | (25) |

Supplementary Table 2: Event numbers per transition from trial data

| To  From | CRPC M0 / M1 lymph | CRPC M1 Bone | CRPC M1 Bone+SRE | CRPC M1 Visceral | PC death | Non-PC death | No event |
| --- | --- | --- | --- | --- | --- | --- | --- |
| M0 / M1 lymph | 463 | 21 |  | 10 | 8 | 33 | 808 |
| M1 bone |  | 1105 |  | 16 | 34 | 21 | 287 |
| M1 visceral |  |  |  | 104 | 4 | 3 | 45 |
| CRPC M0 / M1 lymph |  | 94 |  | 21 | 45 | 21 | 282 |
| CRPC M1 Bone |  |  | 498 | 38 | 218 | 32 | 434 |
| CRPC M1 Bone+SRE |  |  |  | 25 | 285 | 39 | 149 |
| CRPC M1 Visceral |  |  |  |  | 124 | 13 | 77 |

## Patient heterogeneity

Patient heterogeneity is captured by including baseline prognostic factors in the regressions that inform the economic model (namely the survival analysis describing disease progression and the analyses of costs and health related quality of life, HRQoL). The selection of regression covariates is based on existing analyses of metastatic patients^1^ and non-metastatic patients^2^ in the control arm of STAMPEDE which included the following covariates (in addition to presence of bone and visceral metastases which are part of the model structure): presence of lymph node metastases, initial Gleason sum score category, nodal involvement, primary tumour stage, age, prostate-specific antigen (PSA) level, WHO performance status and planned use of radiotherapy (RT). PSA nadir at 24 weeks was not included in the model as this post-baseline measurement would have controlled out some of the effect of treatment.

## Multi-state modelling

The analysis uses the individual patient data from STAMPEDE to estimate all transition probabilities in the state-transition model. Available data for all treatment arms in the STAMPEDE original comparisons (four treatment arms in total) have been used in order to improve the precision of the analysis. However, the cost-effectiveness analysis focuses on the comparison of docetaxel with standard of care (SOC+Doc) to standard of care (SOC) as the addition of zoledronic acid has not been shown to be beneficial.^3^

We have not distinguished in the model between CRPC patients who develop lymph node metastases and CRPC patients who do not have metastases (CRPC M0), as lymph node progression is underdiagnosed in clinical practice (lymph node progression is detected via CT or PET scan and not all practices do this beyond diagnosis). This was reflected in STAMPEDE where we found that very few patients experienced lymph node metastases during the trial.

During the trial metastatic events were classed as lymph node, bone, lung, liver or “other/multiple”. Visceral metastases that were in sites other than lung or liver were therefore included in the “other/multiple” category. The descriptions of the site of metastases for this category were therefore reviewed by the STAMPEDE clinical investigators to determine any that were visceral progression events. These were then grouped with the lung/liver metastases to form the total number of visceral events.

Skeletal related events (SREs) were considered as disease progression only in patients with bone metastases and only after treatment failure, as per clinical advice that other events may not be prostate-cancer related.

## Survival analysis

A series of parametric survival analyses were run to predict within-trial and longer-term event rates. Use of parametric models was necessary, since important clinical events (treatment failure, onset of metastases, death) are not observed for every patient in STAMPEDE due to follow-up limitations, and event rates need to be extrapolated beyond the duration of the trial in the state-transition model. Survival model selection was based on a review of measures of model fit and assessment of visual fit for plausibility and against external data. This process took into consideration both within trial model fit and the plausibility of the extrapolations generated.

### Time to first event

The analysis of time to first event aims to calculate failure-free survival, as per the published clinical analysis ^1^. Table 3 depicts the possible first events that patients can experience, depending on their starting state in the model. As per the clinical analysis time to first event excluded non-prostate cancer death which is modelled separately (see below) but included all other transitions (i.e. transitions 1-4, 6-8, 10-11 from Table 3). A joint model was used for these transitions in order to estimate common treatment and covariate effects (as per the clinical analyses ^1,2^) and to allow estimation of all transitions including those that were relatively rare.

| To  From | CRPC M0 / M1 lymph | CRPC M1 Bone | CRPC M1 Bone+SRE | CRPC M1 Visceral | PC death | Non-PC death |
| --- | --- | --- | --- | --- | --- | --- |
| M0 / M1 lymph | (1) | (2) |  | (3) | (4) | (5) |
| M1 bone |  | (6) |  | (7) | (8) | (9) |
| M1 visceral |  |  |  | (10) | (11) | (12) |

Supplementary Table 3: Possible transitions from starting state (numbered)

A flexible parametric model with 4 degrees of freedom was fitted, similar to the model used in the published clinical analysis ^3^. The fitted model was on the log cumulative odds scale. The model included all baseline covariates (as reported in the section “patient heterogeneity” above), starting state, and a time-dependent treatment effect. An interaction effect between baseline characteristics and metastatic status was included, since we expected that the effect of baseline characteristics on outcomes would vary between non-metastatic and metastatic patients ^1,2^ The inclusion of this interaction improved model fit. Variables denoting whether the transition was a new metastatic progression event (transition 2,3,7) or a transition to PC cancer death (transition 4,8,11) were also included as covariates in the time to first event model, to allow for lower rates of these events.

Alternative models were fitted, where the interaction of baseline covariates with metastatic status was excluded or where the log cumulative hazard instead of the log cumulative odds was modelled. These models were rejected based on AIC criteria and assessment of visual fit of the model predictions generated.

Results from the time to first event model are presented in Table 4 to Table 6. The effect of baseline covariates is broadly consistent with existing analyses of metastatic patients in the control arm of STAMPEDE ^1^. The effect of radiotherapy in non-metastatic patients is also consistent with a published analysis in the control arm of STAMPEDE ^2^. The treatment effect derived from the time to first event model is time-dependent and cannot be presented as a single summary statistic.

Supplementary Table 4: Time to first event model results – all patients

| **Covariate value** | **Odds ratio** | **L95% CI** | **U95% CI** |
| --- | --- | --- | --- |
| Metastatic* at baseline: No | 1.00 |  |  |
| Metastatic* at baseline: Yes | 3.56 | 1.88 | 6.77 |
| Visceral at baseline: No | 1.00 |  |  |
| Visceral at baseline: Yes** | 0.79 | 0.58 | 1.06 |
| M1 lymph at baseline: No | 1.00 |  |  |
| M1 lymph at baseline: Yes** | 1.33 | 1.11 | 1.59 |
| New metastatic progression: No | 1.00 |  |  |
| New metastatic progression: Yes | 0.01 | 0.01 | 0.02 |
| Transition to PC death: No | 1.00 |  |  |
| Transition to PC death: Yes | 0.02 | 0.01 | 0.02 |

A flexible parametric model with 4 degrees of freedom was fitted, as per the published clinical analysis ^3^

* Note that this includes bone or visceral metastases only.

** Note that this an additional effect, above and beyond the effect of having any metastases.

Supplementary Table 5: Time to first event model results –patients who were not metastatic at baseline

| **Covariate value** | **Odds ratio** | **L95% CI** | **U95% CI** |
| --- | --- | --- | --- |
| Lymph node status: N0 | 1.00 |  |  |
| Lymph node status: N+ | 1.64 | 1.29 | 2.10 |
| Lymph node status: NX | 1.78 | 0.57 | 5.57 |
| Tumour stage: <=T2 | 1.00 |  |  |
| Tumour stage: T3 | 1.40 | 0.94 | 2.07 |
| Tumour stage: T4 | 2.06 | 1.27 | 3.33 |
| Tumour stage: TX | 1.38 | 0.68 | 2.78 |
| Gleason-sum score: <=7 | 1.00 |  |  |
| Gleason-sum score: >=8 | 1.47 | 1.12 | 1.92 |
| Gleason-sum score: Unknown | 2.40 | 1.02 | 5.62 |
| Age group: <60 | 1.00 |  |  |
| Age group: 60-64 | 0.73 | 0.53 | 1.00 |
| Age group: 65-69 | 0.67 | 0.49 | 0.92 |
| Age group: >=70 | 0.52 | 0.38 | 0.72 |
| WHO performance status: 0 | 1.00 |  |  |
| WHO performance status: 1 and 2 | 1.09 | 0.79 | 1.50 |
| PSA at randomisation: Quintile 1 | 1.00 |  |  |
| PSA at randomisation: Quintile 2 | 1.00 | 0.73 | 1.37 |
| PSA at randomisation: Quintile 3 | 1.18 | 0.85 | 1.64 |
| PSA at randomisation: Quintile 4 | 1.40 | 1.00 | 1.97 |
| PSA at randomisation: Quintile 5 | 1.89 | 1.18 | 3.02 |
| RT not planned | 1.00 |  |  |
| RT planned | 0.38 | 0.30 | 0.49 |

* A flexible parametric model with 4 degrees of freedom was fitted, as per the published clinical analysis ^3^

Supplementary Table 6: Time to first event model results –patients who were metastatic at baseline

| **Covariate value** | **Odds ratio** | **L95% CI** | **U95% CI** |
| --- | --- | --- | --- |
| Lymph node status: N0 | 1.00 |  |  |
| Lymph node status: N+ | 1.15 | 0.75 | 1.55 |
| Lymph node status: NX | 1.05 | -0.23 | 2.33 |
| Tumour stage: <=T2 | 1.00 |  |  |
| Tumour stage: T3 | 0.94 | 0.42 | 1.46 |
| Tumour stage: T4 | 1.19 | 0.36 | 2.01 |
| Tumour stage: TX | 1.26 | 0.40 | 2.11 |
| Gleason-sum score: <=7 | 1.00 |  |  |
| Gleason-sum score: >=8 | 1.69 | 1.20 | 2.17 |
| Gleason-sum score: Unknown | 1.67 | 0.24 | 3.11 |
| Age group: <60 | 1.00 |  |  |
| Age group: 60-64 | 0.79 | 0.37 | 1.20 |
| Age group: 65-69 | 0.73 | 0.33 | 1.13 |
| Age group: >=70 | 0.61 | 0.21 | 1.02 |
| WHO performance status: 0 | 1.00 |  |  |
| WHO performance status: 1 and 2 | 1.60 | 1.06 | 2.14 |
| PSA at randomisation: Quintile 1 | 1.00 |  |  |
| PSA at randomisation: Quintile 2 | 1.49 | 0.89 | 2.08 |
| PSA at randomisation: Quintile 3 | 1.93 | 1.26 | 2.61 |
| PSA at randomisation: Quintile 4 | 2.11 | 1.43 | 2.79 |
| PSA at randomisation: Quintile 5 | 2.07 | 1.22 | 2.91 |
| RT not planned | 1.00 |  |  |
| RT planned | 0.77 | 0.07 | 1.46 |

* A flexible parametric model with 4 degrees of freedom was fitted, as per the published clinical analysis ^3^

### Time to subsequent events

The allowed subsequent transitions, i.e. transitions following the first event that patients experience, are described in Table 7. This section discusses modelling for the remaining transitions, apart from non-prostate cancer mortality which is discussed in the next section.

Supplementary Table 7: Possible subsequent transitions (numbered)

| To  From | CRPC M0 / M1 lymph | CRPC M1 Bone | CRPC M1 Bone+SRE | CRPC M1 Visceral | PC death | Non-PC death |
| --- | --- | --- | --- | --- | --- | --- |
| CRPC M0 / M1 lymph |  | (13)* |  | (14)** | (15) | (16) |
| CRPC M1 Bone |  |  | (17) | (18)** | (19) | (20) |
| CRPC M1 Bone+SRE |  |  |  | (21)** | (22)*** | (23) |
| CRPC M1 Visceral |  |  |  |  | (24)*** | (25) |

* Covariates on time to treatment failure were highly uncertain and in an unexpected direction (patients who progressed later had a slightly elevated risk of events) and were therefore removed from the model.

** A single model was used for transitions 14, 18, and 21 due to the low numbers of individuals experiencing these events, this model therefore included covariates to allow the rate of these transitions to differ according to current health state.

*** These models did not fit well for the within-trial period and interaction terms between the quartile of progression and treatment allocation were therefore included to allow for the fact that patients who progressed later seemed to derive a benefit from original allocation to docetaxel.

In STAMPEDE only the first of each “type” of progression is documented. Once a distant metastasis progression is reported for a patient - regardless of metastasis site - distant metastases would not be expected to be documented again, even for a different site (note that this does not apply to SREs). This data collection protocol is potentially problematic for patients who are not metastatic at baseline. These patients can experience distant node, bone, or visceral metastasis as a first distant metastasis event, but any subsequent site of distant metastasis would not be reported. Given this and that there was very little follow-up data available beyond the first metastases for these patients; they were censored at the point of the first metastatic event. From this point (i.e. entry to CRPC M1 bone, CRPC M1 bone+SRE or CRPC M1 visceral), rates of transitions are estimated from patients who were metastatic at baseline. This assumes that outcomes amongst metastatic CRPC patients are independent of whether patients were metastatic at the point of initiating hormone therapy, and was supported by the literature ^4^ and clinical opinion. There may also be some unrecorded transitions amongst metastatic CRPC patients; this is because if patients started with bone metastases and then experienced another metastatic bone progression, any subsequent visceral metastases would be missed. This would not be expected to have a large impact on the results as the higher rate of death following visceral metastases would still be included within the analyses.

Subsequent transitions were modelled via a series of separate models which included a covariate for original treatment allocation and for time to treatment failure. The latter was modelled in quartiles but the first and second quartiles (i.e. those patients with the worst prognosis and who therefore progressed most quickly) were combined as these patients had very similar outcomes. The quartiles were defined separately for M0/M1 lymph and M1 patients. Additional features of specific models are recorded in Table 7.

A range of parametric survival models (exponential, generalised gamma, lognormal, and Weibull) were tested and models were selected on the basis of AIC criteria and assessment of visual fit. Results are presented in Table 8 to Table 14. The models are parameterised using an acceleration factor scale for the effect of covariates; the effect column in Table 8 to Table 14 was therefore calculated as one over the acceleration factor estimated from the model, to improve interpretability (so that as for a hazard ratio, values less than 1 indicate improved outcomes, and values in excess of 1 worsened outcomes). Time to progression in either of the two highest quartiles was associated with improved prognosis. Controlling for time to progression, treatment with docetaxel was associated with worsened outcomes post progression (a “catch up effect” was observed) or had little effect.

Supplementary Table 8: Model results for transition 13

| **Covariate** | **Effect size** | **L95% CI** | **U95% CI** |
| --- | --- | --- | --- |
| SOC | 1.00 |  |  |
| SOC+Doc | 1.01 | 0.92 | 1.11 |

Note: A gamma model was fitted.

Note: the effect size column was calculated as one over the acceleration factor estimated from the model, to improve interpretability (so similarly to a hazard ratio, values less than 1 indicate improved outcomes, and values in excess of 1 worsened outcomes). This applies to the effect size columns in the subsequent tables.

Supplementary Table 9: Model results for transitions 14, 18, 21

| **Covariate** | **Effect size** | **L95% CI** | **U95% CI** |
| --- | --- | --- | --- |
| SOC | 1.00 |  |  |
| SOC+Doc | 1.74 | 0.66 | 4.63 |
| Time to progression in Q3 | 0.44 | 0.19 | 1.03 |
| Time to progression in Q4 | 0.23 | 0.09 | 0.61 |
| Health state: M0 CRPC | 1.00 |  |  |
| Health state: M1 CRPC bone | 1.25 | 0.53 | 2.93 |
| Health state: M1 CRPC bone+SRE | 1.74 | 0.68 | 4.47 |

Note: A lognormal model was fitted.

Supplementary Table 10: Model results for transition 15

| **Covariate** | **Effect size** | **L95% CI** | **U95% CI** |
| --- | --- | --- | --- |
| SOC | 1.00 |  |  |
| SOC+Doc | 1.55 | 0.76 | 3.17 |
| Time to progression in Q3 | 0.78 | 0.43 | 1.42 |
| Time to progression in Q4 | 0.38 | 0.17 | 0.86 |

Note: A lognormal model was fitted.

Supplementary Table 11: Model results for transition 17

| **Covariate** | **Effect size** | **L95% CI** | **U95% CI** |
| --- | --- | --- | --- |
| SOC | 1.00 |  |  |
| SOC+Doc | 0.96 | 0.64 | 1.45 |
| Time to progression in Q3 | 0.64 | 0.45 | 0.91 |
| Time to progression in Q4 | 0.44 | 0.30 | 0.65 |

Note: A gamma model was fitted.

Supplementary Table 12: Model results for transition 19

| **Covariate** | **Effect size** | **L95% CI** | **U95% CI** |
| --- | --- | --- | --- |
| SOC | 1.00 |  |  |
| SOC+Doc | 1.20 | 0.93 | 1.56 |
| Time to progression in Q3 | 0.67 | 0.53 | 0.84 |
| Time to progression in Q4 | 0.45 | 0.34 | 0.58 |

Note: A gamma model was fitted.

Supplementary Table 13: Model results for transition 22

| **Covariate** | **Effect size** | **L95% CI** | **U95% CI** |
| --- | --- | --- | --- |
| SOC | 1.00 |  |  |
| SOC+Doc | 1.88 | 1.36 | 2.59 |
| Time to progression in Q3 | 0.62 | 0.48 | 0.79 |
| Time to progression in Q4: SOC | 0.64 | 0.38 | 1.08 |
| Time to progression in Q4: SOC+Dox (interaction effect) | 0.34 | 0.15 | 0.79 |

Note: A gamma model was fitted.

Supplementary Table 14: Model results for transition 24

| **Covariate** | **Effect size** | **L95% CI** | **U95% CI** |
| --- | --- | --- | --- |
| SOC | 1.00 |  |  |
| SOC+Doc | 3.09 | 1.78 | 5.37 |
| Time to progression in Q3 | 0.65 | 0.41 | 1.02 |
| Time to progression in Q4: SOC | 0.47 | 0.20 | 1.09 |
| Time to progression in Q4: SOC+Dox  (interaction effect) | 0.34 | 0.08 | 1.43 |

Note: A lognormal model was fitted.

###

### Time to non-prostate cancer mortality

We anticipated that other-cause mortality would depend only on the patient age. A joint model was hence fitted for all transitions to other-cause mortality, adjusted for patient age at randomisation. However, the model underestimated other-cause mortality in the more severe states (i.e. CRPC states). So, the final other-cause mortality model was also adjusted for membership of the CRPC health states. CRPC patients with bone metastases who had experienced an SRE, and those with visceral metastases had particularly elated rates of non-prostate cancer mortality. This may reflect a tendency for the recorded cause of death to reflect proximal causes of death. Model results for the other-cause mortality model are shown in Table 15.

Supplementary Table 15: Model results for other-cause mortality

| **Covariate value** | **Effect size** | **L95% CI** | **U95% CI** |
| --- | --- | --- | --- |
| Age <60 | 1.00 |  |  |
| Age 60-64 | 1.11 | 0.74 | 1.66 |
| Age 65-69 | 1.06 | 0.71 | 1.57 |
| Age >=70 | 1.59 | 1.11 | 2.28 |
| CRPC M0 | 2.54 | 1.67 | 3.84 |
| CRPC M1 Bone | 2.72 | 1.90 | 3.88 |
| CRPC M1 Bone+SRE | 6.03 | 4.19 | 8.67 |
| CRPC M1 Visceral | 5.43 | 3.26 | 9.04 |

Note: A Weibull model was fitted.

It is unlikely that the within-trial evidence could reliably predict the acceleration in death from other causes beyond the trial period. Trends in other-cause mortality rates among males in the general population were therefore obtained from UK life table data which has been previously analysed using a Gompertz distribution ^5^ and applied from the end of STAMPEDE until the full time horizon for the model. The rate of death predicted by the Gompertz model was inflated by a constant state-specific acceleration factor to the point at which the Gompertz predictions matched the non-prostate cancer death rates observed within STAMPEDE. For states in which other-cause mortality in the trial was lower than the published age-matched all-cause mortality, the rate from UK life tables was used without adjustment. Deaths from prostate cancer were not excluded from the all-cause mortality survival curve and therefore, double counting may occur. However, given that prostate cancer is not listed as a cause of avoidable mortality in published ONS data ^6^, double counting is not a significant concern, or one that could be adjusted for.

### Assessing the validity of model predictions

To assess model fit for the within-trial period plots comparing fitted to predicted failure-free survival (FFS) and overall survival (OS) (Figure 1) were produced, as were cumulative incidence plots for each model transition, in order to examine the observed versus the predicted proportion of patients experiencing the event of interest.

Supplementary Figure 1: Failure-free and overall survival (data and predictions)

The state-transition model will be used make predictions beyond the trial period. It is thus necessary to validate model predictions not only for the trial period, but also to validate longer-term model predictions against available external data from published studies.

We searched the literature for studies with adequately long follow-up and comparable health outcomes for the patient subgroups reflected within STAMPEDE at different stages within the model (M0 hormone-sensitive, M0 CRPC, M1 hormone-sensitive, M1 CRPC). The studies identified were not wholly comparable to the STAMPEDE SOC arm in terms of patient population and did not consistently reflect contemporary treatment practice. Given the uncertainties around the relevance of reported long-term outcome data in the literature to the STAMPEDE population, comparisons with the literature were used to inform additional sensitivity analyses rather than to modify the base case analysis. These comparisons indicated that in non-metastatic patients the model may provide a reasonable approximation of the long-term OS reported in the literature, but underestimate FFS and overestimate post-failure life-expectancy over the long-term compared to the literature. ^7^ ^8^ ^9^ ^10^ For metastatic patients the model may underestimate OS due to an underestimate of FFS over the long term, compared to the literature.^11,12^ The potential for these outcomes to differ in the long-term from the base case model settings were explored in sensitivity analyses as documented in Table 31.

## Quality of life weights

### Data collection

Health-related quality of life (HRQoL) data was collected in STAMPEDE using the EQ-5D patient questionnaire. The EQ-5D is a measure of patient HRQoL that can be used across different diseases and for which general-population preference scores are available that allow patient responses to be converted to HRQoL weights. The EQ-5D questionnaire captures patient HRQoL on five dimensions: mobility, self-care, usual activities, pain/discomfort, and anxiety/ depression. In the version of the EQ-5D used within STAMPEDE each dimension can have 3 levels of responses (EQ-5D-3L): no problem, some problems, and extreme problems. A series of health states are defined by the level recorded for each of the 5 dimensions. To derive health related quality of life weights the responses to the EQ-5D-3L were converted into a single index by applying a set of weights that reflect time trade- off responses from a representative sample of 2,997 noninstitutionalized individuals in the UK.^13,14^

In STAMPEDE quality of life data was collected at baseline and at follow-up visits: every 6 weeks for the first 6 months, then every 12 weeks up until two years, every 6 months up until year 5 and annually thereafter. Data was collected by the research nurse at appropriate clinical visits, but if no clinical visit was scheduled for the patient at that time the nurse was required by the study protocol to organise the completion of the questionnaire by post or by a visit to the patient at home (or in a hospice). Quality of life was initially planned to be collected for the first 700 patients enrolled in the trial, however from protocol version 8.0 (September 2011) the quality of life sub-study was re-opened to all new patients enrolling in the trial. All patients who consented to participate in the quality of life study at baseline were included in the analysis and overall 1,383 patients contributed data to the quality of life analysis. These patients contributed a total of 10,771 EQ-5D response questionnaires over time.

### Statistical analysis

Inevitably, there was a degree of missing quality of life data. Observations were considered as “missing” if the patient was still followed up for vital status within the trial but had not completed a questionnaire at any point between the mid-point between the last scheduled questionnaire and the scheduled questionnaire and the mid-point between the scheduled questionnaire and the next scheduled questionnaire. For example the visit at 30 months was considered as missing if no quality of life form was completed between month 27 and month 33. In order to allow for systematic differences between reported and missing quality of life observations, multiple imputation by chained equations (MICE) ^15^ was used. This was conducted separately for each study arm and using matching to preserve the distribution of the EQ-5D index values (which cannot exceed 1.0, and typically takes a bimodal distribution). The imputation included all potentially prognostic variables available at baseline, timing of quality of life data collection, quality of life data over time, health state over time, death at any time point, and time of death (in line with the “full imputation” approach taken in Rintoul et al. ^16^). Cost data were not included alongside EQ-5D data as the MICE models did not converge when both data sets were imputed simultaneously.

Clinical feedback indicated that important determinants of quality of life were likely to be patient age, WHO status, nodal status, health state and the short-term impacts of chemotherapy which are expected to last for no more than one year. These variables were included in a regression model including repeated measures on each individual included within the analysis.

Generalised estimating equations were used to account for the within-individual correlation in observations. A two-part model was used to account for the large number of observations in which patients recorded an EQ-5D value of 1.0 (full health). The first model uses a logistic regression to predict the probability an individual will record full health. This regression includes all observations on all individuals. The second model predicts the shortfall in quality of life for those who are not at full health and includes observations where individuals recorded an EQ-5D value below 1.0. This second model used a Gamma distribution to model response (as the shortfall is bounded at zero). Other specifications for the model (namely use of the Poisson distribution) were considered but were found to perform worse based upon a measure of goodness of fit (the Quasi-likelihood Information Criterion, QIC). Adequacy of the final model was assessed using a set of tests previously proposed for the assessment of model adequacy in the context of EQ-5D measurements ^17^. The parameter estimates for the models are presented as Table 16. The combined marginal impact of each coefficient is shown in the main manuscript for a specific patient.

Supplementary Table 16****: Quality of life model parameters*****

| **Variable** | **Odds ratio from model 1** | **QOL decrement from model 2** |
| --- | --- | --- |
|  | **Mean (95% CI)** | **Mean (95% CI)** |
| **Constant (odds scale for model 1)** | 0.38 (0.35, 0.40) | 0.34 (0.32, 0.35) |
| **WHO status** |  |  |
| 1 and 2 | 0.60 (0.55, 0.64) | 0.10 (0.08, 0.11) |
| **Age** |  |  |
| ≤60 | - | - |
| 60-64 | 1.22 (1.14, 1.42) | -0.01 (-0.02, 0.01) |
| 65-69 | 1.38 (1.42, 1.48) | -0.04 (-0.05, -0.02) |
| ≥70 | 1.45 (1.37, 1.55) | -0.04 (-0.05, -0.02) |
| **Nodal status** |  |  |
| N0 | - | - |
| N+ | 0.91 (0.86, 0.97) | 0.00 (-0.01, 0.01) |
| NX (unknown) | 1.01 (0.90, 1.13) | -0.01 (-0.03, 0.02) |
| **Treatment** |  |  |
| First year on SOC | 1.03 (0.98, 1.08) | -0.03 (-0.05, -0.02) |
| First year on SOC+Doc | 0.95 (0.87, 1.02) | -0.01 (-0.03, 0.01) |
| **Health state** |  |  |
| Hormone sensitive M0 | - | - |
| Hormone sensitive M1 bone | 0.95 (0.92, 1.00) | 0.01 (0.00, 0.02) |
| Hormone sensitive M1 visceral | 1.00 (0.87, 1.15) | 0.00 (-0.02, 0.03) |
| M0 CRPC | 0.85 (0.77, 0.94) | 0.04 (0.02, 0.07) |
| M1 CRPC bone | 0.73 (0.65, 0.81) | 0.05 (0.03, 0.07) |
| M1 CRPC bone+SRE | 0.48 (0.40, 0.56) | 0.11 (0.09, 0.14) |
| M1 CRPC visceral | 0.46 (0.36, 0.60) | 0.10 (0.06, 0.13) |

* Note that parameters from model 1 and 2 jointly determine the EQ-5D level and should not be interpreted independently.

## **Resource use and costing data**

### Data collection

**STAMPEDE recorded resource use relating to: standard of care treatments, trial treatments, concomitant treatments, radiotherapy, post-progression therapies, surgical interventions, hospital attendances and primary care attendances. We included data on three additional categories of resource use not collected within STAMPEDE but expected to occur in routine practice: (i) scheduled patient monitoring, which would be expected in clinical practice; (ii) end of life care; and (iii) additional costs relating to treatment adverse events. These data were estimated from the literature and clinical opinion.**

**Costs were sourced from public sources and published studies. For generic drugs and pharmaceutical products, prices from the electronic market information (eMIT) tool ^18^ were used where available for the base-case, as these reflect the actual prices paid by NHS hospitals. Prices from the British National Formulary (BNF) ^19^ for these generic drugs were used in a sensitivity analysis.**

**Further details of the costing approach are described by category below.**

### Hormone therapy

**All patients in STAMPEDE received androgen deprivation therapy (ADT) as the standard of care treatment. The method of ADT is an individualised choice but had to be specified prior to patient randomisation. STAMPEDE permitted standard of care of hormone therapy for at least 2 years with gonadotropin-releasing hormone agonists or antagonists or, only between 2006 and 2011 for patients with non-metastatic disease, oral anti-androgens alone. Orchidectomy was an allowable alternative to drug therapy.** Drug costs and dosages were taken from standard sources (Table 17). For the luteinizing hormone-releasing hormone analogues (goserelin, leuprorelin, triptorelin), the average cost of monthly and three-monthly dosing regimens was used. For the administration of injected ADT treatments the average cost of a visit with either a GP or a practice nurse was applied. The use of anti-androgens to prevent tumour “flare” was also costed, using the duration reported in the trial data.

Supplementary Table 17****: Hormone therapy unit costs and dosages****

| **Item** | **Net price** | **Unit / pack size** | **Dosage** | **Source** |
| --- | --- | --- | --- | --- |
| **Goserelin** | £65.00 | 3.6 mg syringe | 3.6 mg, injection every 28 days | BNF, May 2016 **^19^** |
| **Goserelin** | £235.00 | 10.8 mg syringe | 10.8 mg every 12 weeks | BNF, May 2016 |
| **Leuprorelin** | £75.24 | 3.75-mg syringe | 3.75 mg, injection every month | BNF, May 2016 |
| Leuprorelin | £225.72 | 11.25-mg syringe | 11.25 mg every three months | BNF, May 2016 |
| **Triptorelin** | £69.00 | 3-mg vial | 3 mg, injection every 4 weeks | BNF, May 2016 |
| Triptorelin | £207.00 | 11.25-mg vial | 11.25 mg every 3 months | BNF, May 2016 |
| **Bicalutamide** | £1.58 | 50 mg, 28-tab pack | 50 mg tablet, once a day | eMIT, December 2015 |
| **Bicalutamide** | £2.14 | 50 mg, 28-tab pack | 50 mg tablet, once a day | BNF, May 2016 |
| **Cyproterone acetate** | £29.00 | 50 mg, 56-tab pack | 200-300 mg/day - 300mg assumed | BNF, May 2016 |
| **Degarelix** | £260.00 | 2 × 120-mg vials | 240 mg initial dose (2 injections of 120 mg), then 80 mg injection every 28 days | BNF, May 2016 |
| **Degarelix** | £129.37 | 80-mg vial |  | BNF, May 2016 |
| **Administration cost for injected drugs** | £27 | NA | Average cost of visit with GP or practice nurse (11.7 minutes visit length assumed) | PSSRU 2015 |
| **Orchidectomy** | £452.25 | NA | NA | Lord et al ^20^, inflated to 2014–15 prices |

### Trial treatment

**Docetaxel was administered in STAMPEDE at a dose of 75mg/m2 (up to a maximum dose of 160mg) as an intravenous (IV) infusion every 3 weeks, for a maximum of 6 doses. Prednisolone at a dose of 5 mg orally twice daily is administered continuously alongside docetaxel. The acquisition cost of trial treatments (docetaxel, prednisolone) was calculated by multiplying patient-specific doses and numbers of cycles from STAMPEDE, by published unit cost estimates (see Table 18). Patients received different doses of docetaxel according to their body surface area (BSA), which resulted in the use of vials of different volumes. No vial sharing was assumed. The cheapest combination of vials was selected and larger vial sizes were omitted if they could be made up more cheaply with multiple smaller vials.**

**The eMIT prices ^18^ for docetaxel differ significantly versus BNF ^19^ and prices between different versions of eMIT also differ since eMIT documents the average price paid in the NHS hospital-sector for a product over the last 4 months of the reported period. For instance, the price for docetaxel 140mg (20 mg/mL; 7-mL vial) was three times higher than reported in** Table 18**, in a previous NICE appraisal for cabazitaxel ^21^ in which eMIT was accessed in 26/10/2015. Sensitivity analyses around the cost of docetaxel were therefore conducted.**

**The cost of a chemotherapy administration for docetaxel was taken from the National Schedule of Reference Costs 2014-15 ^22^ (see** Table 20**).**

Supplementary Table 18****: Trial treatment unit costs and dosages****

| **Item** | **Net price** | **Unit / pack size** | **Dosage** | **Source** |
| --- | --- | --- | --- | --- |
| **Docetaxel** | £4.92 | 20 mg/mL; 1-mL vial | 75mg/m^2^ every 3 weeks | eMIT, December 2015, STAMPEDE protocol |
| **Docetaxel** | £12.47 | 20 mg/mL; 4-mL vial | 75mg/m^2^ every 3 weeks | eMIT, December 2015, STAMPEDE protocol |
| **Docetaxel** | £17.77 | 20 mg/mL; 7-mL vial | 75mg/m^2^ every 3 weeks | eMIT, December 2015, STAMPEDE protocol |
| **Docetaxel** | £138.33 | 10 mg/mL; 2-mL vial | 75mg/m^2^ every 3 weeks | BNF May 2016, STAMPEDE protocol |
| **Docetaxel** | £454.53 | 10 mg/mL; 8-mL vial | 75mg/m^2^ every 3 weeks | BNF May 2016, STAMPEDE protocol |
| **Prednisolone** | £0.24 | 5 mg, 28-tab pack | 5 mg twice daily continuously with docetaxel | eMIT, December 2015, STAMPEDE protocol |
| **Prednisolone** | £1.24 | 5 mg, 28-tab pack | 5 mg twice daily continuously with docetaxel | BNF May 2016, STAMPEDE protocol |

### Concomitant treatments

**Patients received additional care or medications provided concomitantly with their trial treatment which were recorded at trial entry. For the economic analysis we only included concomitant medication wherever the indication for receiving concomitant treatment included the terms “urinary”, “bone” or “prostate”. Unit costs for these drugs were taken from standard sources, and dosing information was based on standard recommended doses.**

### Post-progression therapies

**Following progression, patients in STAMPEDE received different therapies** at the discretion of the treating physician**. These included drugs, procedures and radiotherapy.** Post-progression drug costs are reported in Table 19 and associated administration costs in Table 20, procedure costs **are reported in** Table 21 and radiotherapy costs in Table 23. Table 22 documents the **mean treatment duration for post-progression drugs.**

**Abiraterone, cabazitaxel, enzalutamide and radium-223 dichloride are subject to confidential pricing agreements which include discounts to the NHS, which have changed over time. These are the subject of sensitivity analyses (see below) as for commercial reasons, only very limited details relating to the pricing agreements are in the public domain.**

**As information on specific doses and durations for post-progression therapies was not generally available from the trial data, this information was obtained from their recommended dosages as detailed in public sources (e.g. BNF or product SPC) and relevant pivotal trials. Clinical opinion was used where the information was not available from the literature.** For treatments where the dose is dependent on BSA, the average patient BSA in STAMPEDE was used to calculate required dosages. No vial sharing was assumed for **post-progression** chemotherapy treatments administered as an intravenous infusion (cabazitaxel, mitoxantrone, carboplatin and etoposide); the cheapest combination of vials was selected.

**RT provided post-progression in STAMPEDE falls into the following categories: RT to prostate, RT for spinal cord compression, RT to bone metastases, and palliative RT. Detailed data on the use of RT have been collected within STAMPEDE. Radiotherapy has been costed based on the** dose-fractionation schedule details from the trial data and the costs for radiotherapy preparation and radiotherapy delivery from NHS reference costs 2014-15 (see Table 23)**. The radiotherapy preparation cost is applied once per RT schedule and the radiotherapy delivery cost is applied every time a** radiotherapy **fraction** is administered. **Upfront use of radiotherapy was also recorded within STAMPEDE, however as these costs were found to be only marginally lower in the docetaxel arm (by about £30), and we did not expect a difference in the use of upfront radiotherapy across study arms, these costs were excluded from the model.**

Supplementary Table 19****: Post-progression drug unit costs and regimens****

| **Item** | **Net price** | **Unit / pack size** | **Dosage used for costing** | **Source** |
| --- | --- | --- | --- | --- |
| **Abiraterone acetate** | £2,930.00 | 250 mg; 120-tab pack | 1 g once daily | BNF, May 2016 |
| **Abiraterone acetate** | £2,300.00 | 250 mg; 120-tab pack | 1 g once daily | NICE TA387, pricing arrangement |
| **Cabazitaxel** | £3,696.00 | 40 mg/mL; 1.5-mL vial | 25 mg/m**^2^** as IV infusion every 21 days + predisone 10 mg daily | BNF, May 2016 |
| **Enzalutamide** | £2,734.67 | 40 mg; 112-cap pack | 160 mg once daily | BNF, May 2016 |
| **Enzalutamide** | £2,146.72 | 40 mg; 112-cap pack | 160 mg once daily | Based on NICE TA387 ^23^, pricing arrangement |
| **Mitoxantrone** | £31.51 | **2 mg/mL** ; 10-mL vial | 14 mg/m**^2^** of body surface area, every 21 days | eMIT, December 2015 |
| **Mitoxantrone** | £100.00 | **2 mg/mL** ; 10-mL vial | 14 mg/m**^2^** of body surface area, every 21 days | BNF, May 2016 |
| **Carboplatin** | £3.57 | 10 mg/mL; 5-mL vial | 400 mg/m**^2^**, as IV infusion every 4 weeks | eMIT, December 2015 |
| **Carboplatin** | **£7.62** | **10 mg/mL, 15-mL vial** | 400 mg/m**^2^**, as IV infusion every 4 weeks | eMIT, December 2015 |
| **Carboplatin** | £20.00 | 10 mg/mL; 5-mL vial | 400 mg/m**^2^**, as IV infusion every 4 weeks | BNF, May 2016 |
| **Carboplatin** | **£50.00** | **10 mg/mL, 15-mL vial** | 400 mg/m**^2^**, as IV infusion every 4 weeks | BNF, May 2016 |
| **Etoposide** | £6.39 | etoposide 20 mg/mL, 5-mL vial | 120 mg/m**^2^** , as IV infusion on days 1, 3, and 5 of each cycle - cycle length 21 days | eMIT, December 2015 |
| **Etoposide** | £12.15 | etoposide 20 mg/mL, 5-mL vial | 120 mg/m**^2^** , as IV infusion on days 1, 3, and 5 of each cycle - cycle length 21 days | BNF, May 2016 |
| **Pamidronate** | £80.00 | 9 mg/mL, 10-mL vial | 90mg IV infusion, every 4 weeks | BNF, May 2016 |
| **Denosumab*** | £183.00 | 60 mg/mL, 1-mL prefilled syringe | 60 mg, injection every 6 months | BNF, May 2016 |
| **Dexamethasone** | £58.27 | 3 mg, 100-tab pack | 6 mg daily | eMIT, December 2015 |
| **Dexamethasone** | £78.00 | 3 mg, 100-tab pack | 6 mg daily | BNF, May 2016 |
| **Diethylstilbestrol** | £114.12 | 1 mg; 28 tablets | 1–3 mg daily; 2mg assumed | BNF, May 2016 |
| **Radium 223** | £24,240 | 50 kbq**/kg of body weight every 4 weeks for 6 injections | Not applicable - average cost of a course of treatment estimated by manufacturer | NICE Technology Appraisal 376 ^24^ |
| **Strontium-89; drug cost** | £1,710.00 | per fraction of 150mbq*** | 148 MBq IV injection, not more frequently than every 90 days | University Hospital Birmingham - Nuclear Medicine department (TRAPEZE study) ^25^ |
| **Strontium-89; administration** | £443.00 | per administration | 148 MBq IV injection, not more frequently than every 90 days |  |
| **Zoledronic Acid** | £2.80 | 800 micrograms/mL, 5-mL (4-mg) vial | 4mg every 3 weeks | eMIT, December 2015 |
| **Zoledronic Acid** | £174.14 | 800 micrograms/mL, 5-mL (4-mg) vial | 4mg every 3 weeks | BNF, May 2016 |

**Note: Hormone therapy and docetaxel were also administered as post-progression therapies; their costs are included in Table 17 and Table 18**

*** For denosumab, the cost of Prolia (Amgen) is assumed**

**** kbq = kilobecquerel; unit of radioactivity**

***** mbq = megabecquerel; unit of radioactivity**

Supplementary Table 20****: Chemotherapy administration costs****

| **Item** | **Cost** | **HRG code** | **Source** |
| --- | --- | --- | --- |
| **Chemotherapy delivery cost for docetaxel, cabazitaxel, mitoxantrone, carboplatin, zoledronic acid and pamidronate** | £239.12 | SB12Z | NHS Reference Costs 2014-15 ^22^ |
| **Chemotherapy delivery cost for etoposide, carboplatin & etoposide, etoposide & mitoxantrone; First attendance** | £389.41 | SB14Z | NHS Reference Costs 2014-15 |
| Cost for delivery of subsequent elements of a chemotherapy cycle* | 326.46 | SB15Z | NHS Reference Costs 2014-15 |

***Applies to etoposide monotherapy or combination therapies including etoposide only. For these regimens, HRG SB14Z is used for the first attendance and the HRG SB15Z cost is applied to each subsequent chemotherapy delivery.**

Supplementary Table 21****: Post-progression procedures unit costs****

| **Item** | **Cost** | **Details** | **Source** |
| --- | --- | --- | --- |
| Colostomy | £6,189.7 | Weighted average of Colon Procedures HRGs (FZ75C-FZ76D) | NHS Reference Costs 2014-15 ^22^ |
| Nephrostomy | £958.72 | Weighted average of Nephrostomy HRGs (YL10Z-YL12Z) | NHS Reference Costs 2014-15 |
| Surgery for spinal cord compression | £11,139.47 | Spinal Cord Conditions with Interventions HRG (HC28H) | NHS Reference Costs 2014-15 |
| Surgery bowel obstruction | £6,189.7 | Weighted average of Colon Procedures HRGs (FZ75C-FZ76D) | NHS Reference Costs 2014-15 |
| Transurethral Prostate Resection Procedures (TURP) | £2,670.20 | Weighted average of TURP HRGs (LB25D-LB25F) | NHS Reference Costs 2014-15 |
| Ureteric stents | £958.72 | Weighted average of Ureteric Stent HRGs (YL10Z-YL12Z) | NHS Reference Costs 2014-15 |
| Urinary catheter | £362.46 | Bladder Catheter HRG (LB18Z) | NHS Reference Costs 2014-15 |

Supplementary Table 22****: Mean treatment duration for post-progression therapies****

| **Drug** | **Mean treatment duration** | **Source** |
| --- | --- | --- |
| **Docetaxel** | 9.5 cycles | TAX327 study ^26^ |
| **Abiraterone** | 13.8 months pre-chemotherapy  8 months post-chemotherapy | COU-AA-302 study ^12^  COU-AA-301 study ^27^ |
| **Enzalutamide** | 15.8 months pre-chemotherapy  8.3 months post-chemotherapy | PREVAIL study ^28^  AFFIRM study ^29^ |
| **Cabazitaxel** | 6 cycles | TROPIC study ^30^ |
| **Mitoxantrone** | 4 cycles | TROPIC study ^30^ |
| **Carboplatin** | 6 cycles | Clinical opinion |
| **Etoposide** | 6 cycles | Clinical opinion |

Supplementary Table 23****: Radiotherapy unit costs****

| **Item** | **Cost** | **Description** | **Details** | **Source** |
| --- | --- | --- | --- | --- |
| **Radiotherapy preparation** | £767.17 | Applied once per radiotherapy schedule | Weighted average of HRG cost for complex conformal radiotherapy (SC51Z- SC52Z) | NHS Reference Costs 2014-15 |
| **Radiotherapy delivery** | £127.81 | Per radiotherapy fraction administered | Weighted average of HRG cost for megavoltage machine radiotherapy delivery (SC22Z- SC23Z) | NHS Reference Costs 2014-15 |

### Hospital and primary care service use

**Data on unscheduled prostate cancer-related outpatient appointments, inpatient stays and GP visits were collected within STAMPEDE. Unit costs for outpatient appointments and inpatient stay were obtained from the NHS reference costs 2014-2015.^22^ Unit cost for GP visits were obtained from the Personal Social Services Research Unit’s (PSSRU) unit costs 2015 (Table 24).^31^ For unplanned in-patient hospital attendances, costs were calculated as follows: if the patient stayed in hospital 0-1 days then the short-stay HRG cost was used; for 2 days or more the long-stay HRG was used. If the patient’s hospital stay exceeded the HRG average trim point (in days), then the long-stay HRG was used and the additional days were costed as excess bed days.**

Supplementary Table 24****: Hospital and primary care services unit costs****

| **Item** | **Cost** | **Description** | **Details** | **Source** |
| --- | --- | --- | --- | --- |
| **Unplanned in-patient days** | £614 | Non-elective short stay | Weighted average cost of non-elective short stay HRGs - Kidney, Urinary Tract or Prostate Neoplasms, without Interventions (LB06N-LB06S) | NHS Reference Costs 2014-15 ^22^ |
|  | £3,316 | Non-elective long stay | Weighted average cost of non-elective long stay HRGs - Kidney, Urinary Tract or Prostate Neoplasms, without Interventions (LB06N-LB06S) | NHS Reference Costs 2014-15 |
|  | 25 days | Trim point for Non-elective long stay HRGs | Trim point for Non-elective long stay HRGs (LB06N-LB06S) - weighted average of days | NHS Reference Costs 2014-15 |
|  | £288 | Excess bed day | Weighted average cost - Kidney, Urinary Tract or Prostate Neoplasms, without Interventions (LB06N-LB06S) | NHS Reference Costs 2014-15 |
| **Unplanned out-patient visits** | £99 | Urology outpatient attendance cost | Service code 101 | NHS Reference Costs 2014-15 |
| **Unplanned visits to GP** | £65 | Per patient contact lasting 17.2 minutes | Used for unplanned visits | PSSRU 2015 |
|  | £45 | Per patient contact lasting 11.7 minutes | Used for visits for hormone therapy administration | PSSRU 2015 |
| **Nurse (GP practice)** | £47 | Per hour of face-to-face contact | Used for visits for hormone therapy administration | PSSRU 2015 |

###

### Adverse events

**Neutropenia and febrile neutropenia rates were higher in the SOC+Doc arm of STAMPEDE than in the SOC arm (15% and 12% respectively in the docetaxel arm versus 1% and 0% in the control arm) ^3^. The hospitalisations associated with (febrile) neutropenia should already be reflected within the STAMPEDE data, however the NHS reference cost used does not reflect the use of high cost drugs to treat neutropenia, or the higher costs of hospitalisation for an extreme event of this kind. This was therefore reflected by assuming an extra drug cost per episode of neutropenia of** £128 (HRG code XD25Z (Neutropenia Drugs, Band 1)). For febrile neutropenia, the additional cost assigned per episode was based on the difference between febrile neutropenia HRG (NHS reference costs 2012-2013; NEI_L: Weighted average of PA45Z, £4,679) (TA377 ^32^, in 2014-15 values) and the non-elective long stay cost assumed in the model for other hospitalisations (weighted average of non-elective long stay HRGs LB06N-LB06S, £3,316).

### Patient monitoring

**Scheduled patient monitoring (which includes outpatient visits, scans and laboratory tests) that would be expected in clinical practice has not been captured in the STAMPEDE data collection. This was estimated from the literature and, where no directly relevant data was identified from the literature, on the basis of clinical opinion. Patients with hormone sensitive disease (either non-metastatic or metastatic) were assumed to have the same monitoring resource use as used for active surveillance (based on Ramsey et al ^33^), adjusted via clinical opinion to reflect the increased severity of patients in STAMPEDE (**Table 25**). Castrate-resistant patients were assumed to receive patient monitoring based on NICE TA387 ^23^, also validated by clinical opinion (**Table 26**) who confirmed that the onset of CRPC is associated with more intense patient monitoring. For patients receiving investigational treatment with docetaxel or other chemotherapy, or abiraterone or enzalutamide, monitoring-related resource use for the treatment period was informed by clinical opinion (**Table 26**). The experts noted that monitoring for abiraterone or enzalutamide was likely to be somewhat less intense but agreed that reflecting this difference was unlikely to have a meaningful impact. The unit costs associated with patient monitoring are presented in** Table 27**.**

Supplementary Table 25****: Resource use associated with monitoring - hormone sensitive patients****

|  | **Year 1** | **Year 2-5** |
| --- | --- | --- |
| Resource use | 4 doctor-led outpatient appointments | 2 doctor-led outpatient appointments |
|  | 4 PSA tests | 2 PSA tests |
|  | 1 CT scan | 2 CT scans |
|  | 1 bone scan | 2 bone scans |
|  | 1 MDT meeting | - |
| Annual cost | £684 | £538 |
| 3-month cost | £171 | £135 |

**CRPC, Castrate-resistant prostate cancer; PSA, prostate-specific antigen; CT, computed tomography; MDT, Multi-disciplinary team**

Supplementary Table 26****: Resource use associated with monitoring - patients with CRPC and patients**** r****eceiving life-extending therapy****

|  | Receiving docetaxel or other chemotherapy or abiraterone / enzalutamide | | Castrate-resistant prostate cancer (CRPC) patients | |
| --- | --- | --- | --- | --- |
|  | % of patients requiring | Frequency in a 3-month period | % of patients requiring | Frequency in a 3-month period |
| Out-patient visit | 100 | 4 | 100 | 3 |
| CT scan | 80 | 1 | 60 | 1 |
| Radiographic /MRI scan | 5 | 1 | 10 | 1 |
| Bone scan | 50 | 1 | 50 | 1 |
| ECG | 10 | 1 | 10 | 1 |
| Full blood count | 100 | 4 | 100 | 3 |
| Liver function test | 100 | 4 | 100 | 3 |
| Kidney function test | 100 | 4 | 100 | 3 |
| PSA | 100 | 4 | 100 | 3 |
| Annual cost | £2,256 | | £1,764 | |
| Total cost for 3 months | £564 | | £441 | |

**CRPC, Castrate-resistant prostate cancer; CT, computed tomography; MRI, magnetic resonance imaging; ECG, electrocardiogram; PSA, prostate-specific antigen**

Supplementary Table 27****: U****nit costs associated with patient monitoring

| **Resource item** | **Unit cost, £** | **Details** | **Source** |
| --- | --- | --- | --- |
| Out-patient visit | 99 | Total Outpatient Attendances, code 101 | NHS Reference Costs 2014-15 |
| CT scan | 109 | Weighted average of HRGs for CT scan (RD22Z- RD27Z) | NHS Reference Costs 2014-15 |
| Radiographic/MRI scan | 147 | Weighted average of HRGs for CT scan (RD03Z- RD06Z) | NHS Reference Costs 2014-15 |
| Bone scan | 59 | HRG RD50Z | NHS Reference Costs 2014-15 |
| ECG | 65 | HRG RD51A | NHS Reference Costs 2014-15 |
| Full blood count | 3 | HRG DAPS08 | NHS Reference Costs 2014-15 |
| Liver function test | 2 | HRG DAPS03 | NHS Reference Costs 2014-15 |
| Kidney function test | 2 | HRG DAPS03 | NHS Reference Costs 2014-15 |
| PSA | 2 | HRG DAPS03 | NHS Reference Costs 2014-15 |
| Cancer MDT Meeting | 110.5 | CMDT_Oth | NHS Reference Costs 2014-15 |

**CT, computed tomography; MRI, magnetic resonance imaging; ECG, electrocardiogram; PSA, prostate-specific antigen; TRUS, Transrectal Ultrasound, MDT, Multi-disciplinary team**

### End of life care

**The cost of end of life care for prostate cancer patients is estimated to be £6,931 according to Hounsome et al ^34^, based on Hospital Episode Statistics for urological cancers for the period 2006-08. Round et al ^35^ estimated the health care cost for people with prostate cancer at the end of life to be £6,687, based on a systematic review of data from UK-based studies and publicly available data sets. Although both estimates are very similar, for our analysis we used the end of life cost based on Round et al, as a more recent estimate.**

### Statistical analysis

As for quality of life data, there was a degree of missing data in the cost data and missing data were imputed using MICE ^15^. Data were imputed for any observations beyond the point of the last follow-up form that collected cost data up until the last follow-up for vital status. The imputation included all potentially prognostic variables available at baseline, treatment group, health state over time, costs at other time points, death at any time point, and time of death (in line with the “full imputation” approach taken in Rintoul et al. ^16^). EQ-5D data were not included alongside cost data as the MICE models did not converge when both data were imputed simultaneously. Costs were not imputed separately by trial arm as again the models did not converge.

Clinical feedback indicated that important determinants of costs were likely to be patient age, WHO status, nodal stage, health state and the short-term impacts of chemotherapy (which are expected to last for no more than one year). These variables were therefore considered as potential determinants of costs within the analysis.

Data on upfront docetaxel acquisition, administration and monitoring and adverse event costs could be simply assigned to patients within the model according to their study arm and baseline characteristics (docetaxel costs were found to differ by WHO status and age, see Table 28).

Monitoring costs were assigned according to patient health state and treatment status.

In order to facilitate extrapolation of the remaining costs collected within STAMPEDE, analyses were conducted to estimate the impact of health state on costs. The purpose of this analysis was to allow the increasing proportion of individuals in the more severe health states over the lifetime time horizon of the model (and differences in this across study arms) to appropriately impact on lifetime cost estimates. Two analyses were conducted: one of long-term management costs which included: **hormone therapy and concomitant therapy; post-progression drugs (excluding life-extending drugs), procedures and radiotherapy; and unscheduled primary care, outpatient and inpatient visits considered related to prostate cancer.** Costs were estimated in 4-month intervals, and these data were analysed using generalised estimating equations to account for the within-individual correlation in observations. A Poisson model was found to fit the data best and is able to characterise the nature of the cost distribution which is bounded by zero. A gamma model was also considered but found to perform worse based upon a measure of goodness of fit (the Quasi-likelihood Information Criterion, QIC). Adequacy of the final model was assessed using a set of tests previously proposed by Basu and Manca ^17^. The parameter estimates for the models are presented in the main text.

The second analysis focused on life-extending drugs which were defined in the STAMPEDE clinical **analysis ^3^ to comprise docetaxel, abiraterone, enzalutamide, cabazitaxel and radium-223. These drugs are predominantly administered to patients with metastatic CRPC. Although total rates of use of life-extending therapies amongst CRPC patients were similar across study arms, the types of therapy differed. Patients randomised to the SOC arm were more likely to have received docetaxel as a first life-prolonging therapy, whereas patients randomised to the SOC+Doc arm were more likely to have received abiraterone or enzalutamide. This likely reflected the licenses for abiraterone and enzalutamide operating at the time as during the majority of the STAMPEDE follow-up period abiraterone was licensed for use in those who had failed chemotherapy. This appears to have resulted in high use of abiraterone/enzalutamide in the docetaxel arm (where patients had failed upfront chemotherapy by definition at onset of CRPC) and much lower use for those in the SOC arm where patients were chemotherapy naïve at the point of onset of CRPC. There is a limited volume of data on life-extending therapy as it is primarily restricted to patients who have failed hormone therapy and are metastatic, thus reducing the scope for exploring the impact of different patient characteristics. These data were therefore analysed by calculating the mean cost of life-extending therapy by key variables: randomised treatment, health state, and time spent in each health state. Time periods of 0-1 year, 1-2 years and 3+ years spent in the health state were used to stratify costs as there was very little data beyond year three. Given the sparsity of data amongst patients who had spent three or more years in a health state, data was pooled across study arms from the third year of time in any state for the remainder of the model. This analysis is presented in the main text.**

## Reflecting current treatment practice in the UK

**Abiraterone and enzalutamide are now licensed and recommended by NICE for use in chemotherapy-naïve patients. ^23^ ^32^ These therapies are now likely to be the first-line treatment choice for the majority of patients with metastatic CRPC who receive life-extending therapy.^36^ Given this, for the base case analysis we assumed that the high use of abiraterone and enzalutamide observed in the SOC+Doc arm of STAMPEDE would also be observed in the SOC arm. In this analysis the life-extending therapy costs observed in the SOC+Doc arm were therefore applied to the SOC arm.**

**Higher use of abiraterone and enzalutamide in the SOC arm is expected to improve outcomes as well as incurring costs.^12^ ^28^ This was included in the model as follows. Firstly, we estimated the additional proportion of M1 CRPC patients who received abiraterone or enzalutamide as their first life-extending therapy on the SOC+Doc arm compared to the SOC arm. This was estimated to be 20% based upon all patients who receive abiraterone/enzalutamide in the M1 CRPC states (M1 Bone, M1 Bone+SRE and M1 Visceral). We then applied the hazard ratio on OS (0.81, 95% CI 0.70-0.93) from the COU-AA-302 trial^12^ to the time from onset of metastatic CRPC to death for the 20% of patients expected to switch to abiraterone plus enzalutamide in the SOC arm. COU-302 compared abiraterone to placebo in chemotherapy naïve men with metastatic CRPC. Some patients residing in the M0/M1 lymph node CRPC state also received abiteraterone or enzalutamide within STAMPEDE. A sensitivity analysis was therefore run to reflect the higher use in the SOC arm expected in practice in these patients.**

## Evaluation of the disease model

**The model was evaluated using a patient level simulation approach ^37^. This allowed time-dependences in the rate of clinical events to be reflected for all transitions. The model was evaluated 40 times for each of the 2962 patients within STAMPEDE (i.e. 118,480 times) assuming that all patients had been allocated to SOC and then 118,480 times assuming all patients had been allocated to SOC+Doc. Multiple runs indicated that the results were stable with this number of simulations.^37^**

## Sensitivity analyses conducted

**The full list of sensitivity analyses, the rationale for each analysis and the method of implementation are provided as** Table 31**. The full results of the sensitivity analyses are presented as** Table 32**. A probabilistic sensitivity analysis was also conducted. This involved drawing 1,000 values for the parameters of interest. For each set of parameter simulations the patient level simulation was then run in full and the average results stored. Multivariate normal distributions were used for parameters derived from the regression analyses. Distributions for the additional parameters estimated from STAMPEDE were obtained via bootstrapping, and gamma distributions were used for cost parameters derived from the literature (i.e. monitoring and end of life costs). For the monitoring costs no measure of uncertainty was available as these costs were based on guidelines and expert opinion. For these parameters we therefore assumed that the standard error was equal to half the value of the mean.**

Supplementary Table 31: Sensitivity analyses conducted

| **Sensitivity analysis** | **Rationale** | **Implementation** |
| --- | --- | --- |
| **Analyses relating to disease progression rates** |  |  |
| **Include data from meta-analysis of docetaxel trials** | **A recent meta-analysis^38^ found two additional trials of docetaxel in metastatic patients, and three additional trials of docetaxel in non-metastatic patients. This analysis reflects the estimates of treatment effect on the FFS and OS endpoints estimated from this meta-analysis.** | **The ratio of the hazard ratio observed in the meta-analysis to that observed in STAMPEDE was applied to the docetaxel FFS and OS curves estimated from the model. This gave an adjusted estimate of time spent pre and post-failure. The distribution across different CRPC states was assumed as per the base case.** |
| **No overall survival benefit associated with docetaxel in non-metastatic patients** | **The survival benefit observed to date in STAMPEDE and the meta-analysis described above is not statistically significant and the analyses to date are considered underpowered. This analysis therefore explores the possibility that docetaxel does not extend OS in non-metastatic patients.** | **Increase rate of metastases amongst patients with non-metastatic CRPC in patients randomised to SOC+Doc.** |
| **Worsened prognosis for patients with non-metastatic disease following onset of CRPC** | **The model predicted better long-term outcomes than observed in previous studies for individuals with non-metastatic CRPC, use of alternative parametric distributions reflecting a more severe prognosis in the long-term were therefore tested.** | **Weibull model used following onset of CRPC for non-metastatic patients (applied for transitions 13, 14, 15).** |
| **Improve FFS outcomes** | **The model predicted worse long-term failure-free survival outcomes than observed in previous studies for individuals with non-metastatic and metastatic hormone-sensitive disease.** | **A flexible parametric model was fitted which produced more optimistic long-term extrapolations for FFS.** |
| **Modelling of non-prostate cancer mortality in the extrapolation period** | **The base case applies all-cause mortality data from the general population inflated by a constant multiplicative factor to reflect the higher rate of non-prostate cancer-related deaths observed in STAMPEDE patients compared to the general population. This sensitivity analysis assumes that the inflation is via a constant additive risk rather than relative risk to allow for the fact that the additional risk observed in STAMPEDE may not continue to rise as patients’ age.** | **The gompertz distribution is modified to reflect an additive risk.** |
| **Analyses relating to treatment pathway** |  |  |
| **Assume all non-metastatic patients receive radiotherapy** | **There is now compelling data to support use of radiotherapy as part of standard care in hormone sensitivie patients with node-negative non-metastatic disease, and some data to support use of radiotherapy in patients with node-positive non-metastatic disease. This will improve the prognosis of non-metastatic patients and was therefore reflected within the model.** | **The covariate indicating whether or not radiotherapy is planned in the analysis of FFS is switched “on” for all patients with non-metastatic disease at baseline and the model re-run.** |
| **Remove costs of abiraterone or enzalutamide when administered after a prior course of abiraterone or enzalutamide** | **Available clinical data does not support the effectiveness of a second course of abiraterone or enzalutamide after failure of a first course and this use of these treatments is unlikely to be funded by the NHS.** | **Cost of repeat use of abiraterone/enzalutamide set to zero.** |
| **CRPC treatments differ according to original treatment allocation, as observed in STAMPEDE** | **Test sensitivity of cost-effectiveness results to patients receiving treatments as observed in STAMPEDE (i.e. lower rate of abiraterone/enzalutamide use in SOC arm).** | **Use observed arm-specific costs of life-extending therapies, remove adjustment of SOC outcomes for increased use of abiraterone/enzalutamide.** |
| **Adjust outcomes in SOC arm for M0/M1 lymph node CRPC patients who might be expected to receive abiraterone/enzalutamide under current practice** | **Test sensitivity of results to improving SOC outcomes to reflect higher use of abiraterone and enzalutamide.** | **Adjust for difference (13%) in use of abiraterone/enzalutamide in M0/M1 lymph node state between docetaxel and SOC using data from COU-302 trial.** |
| **Analyses relating to costs** |  |  |
| **Patient access scheme costs applied for abiraterone and enzalutamide** | **Reflect minimum likely discounts received by NHS.** | **Unit costs and durations adjusted.** |
| **Patient access scheme costs applied for abiraterone and enzalutamide and 20% discount applied for other branded life-extending therapies where no information is available regarding discounts (radium-223, cabazitaxel)** | **Reflect more probable scenario regarding discounts received by the NHS.** | **Unit costs and durations adjusted** |
| **BNF prices used in place of eMIT prices** | **Test sensitivity of results to removal of typical discounts NHS receives on generic products.** | **Replace all eMIT costs with BNF costs** |
| **Increase subsequent therapy costs to account for additional missing data** | **Post-progression therapy CRFs were completed each time a post-progression therapy was initiated. It is possible that even though a patient was retained within a trial and completed other follow-up forms that subsequent therapy forms were not completed every time these treatments were administered. We therefore tested the sensitivity of the results to assuming additional missing data for life-extending therapies.** | **Consultation with the STAMPEDE data management team indicated that the degree of missing data was likely to increase the longer patients had resided in a health state. We therefore assumed that 10%, 20% and 30% of life-extending therapy lines initiated were missed in the first, second, and third year in each state, respectively.** |

Supplementary Table 32: Sensitivity analyses - results

|  | **Impact on ICER (£/QALY)** | |
| --- | --- | --- |
|  | **Non-metastatic patients** | **Metastatic patients** |
| **Base case** | **Dominant** | **5,514** |
| **Analyses relating to transition rates** |  |  |
| **Include data from meta-analysis of docetaxel trials** | **8,827** | **8,295** |
| **No overall survival benefit associated with docetaxel in non-metastatic patients** | **Dominant** | **Only affects non-metastatic patients** |
| **Worsened prognosis for patients with non-metastatic disease following onset of CRPC** | **Dominant** | **Only affects non-metastatic patients** |
| **Improve FFS outcomes** | **Dominant** | **4,911** |
| **Modelling of non-prostate cancer mortality in the extrapolation period** | **Dominant** | **5,597** |
| **Analyses relating to treatment pathway** |  |  |
| **Assume all non-metastatic patients receive radiotherapy** | **Dominant** | **Only affects non-metastatic patients** |
| **Remove costs of abiraterone or enzalutamide when administered after a prior course of abiraterone or enzalutamide** | **Dominant** | **5,513** |
| **No life extending therapy costs beyond 2 years in any health state** | **2,993** | **5,251** |
| **CRPC treatments differ according to original treatment allocation, as observed in STAMPEDE** | **13,299** | **18,342** |
| **Adjust outcomes in SOC arm for M0/M1 lymph node CRPC patients who might be expected to receive abiraterone/enzalutamide under current practice** | **Dominant** | **Only affects non-metastatic patients** |
| **Analyses relating to costs** |  |  |
| **Patient access scheme costs applied for abiraterone and enzalutamide** | **1,702** | **5,987** |
| **Patient access scheme costs applied for abiraterone and enzalutamide and 20% discount applied for other branded life-extending therapies where no information is available regarding discounts (radium-223, cabazitaxel)** | **2,290** | **6,209** |
| **BNF prices used in place of eMIT prices** | **10,610** | **13,868** |
| **Increase subsequent therapy costs to account for additional missing data** | **Dominant** | **5,231** |

## STAMPEDE investigators and trial committees

**STAMPEDE investigators:**

**Key:** Site (N1/N2: Site Principle Investigator; Other randomising consultants) where N1 = accrual from trial start to end of recruitment to docetaxel and zoledronic acid arms (Oct‑2005 to Mar‑2013) and N2 = accrual from trial start to data freeze (Oct‑2005 to 13‑May‑2015)

**UNITED KINGDOM**

**Aberystwyth, Bronglais General Hospital**(3/4: Sajid Durrani)

**Ashford, William Harvey Hospital**(4/11: Carys Thomas; Natasha Mithal)

**Aylesbury, High Wycombe & Stoke Mandeville Hospital**(6/12: Ami Sabharwal; Philip Camilleri, Christopher Alcock, Andrew Protheroe, Joanne Brady)

**Ayr, Ayr Hospital**(27/45: Hilary Glen; Jawaher Ansari, Rana Mahmood)

**Barnet, Barnet General Hospital**(8/17: Ursula McGovern; Andrew Eichholz)

**Barnstaple, North Devon District Hospital**(15/25: Denise Sheehan)

**Basingstoke, Basingstoke and North Hampshire Hospital**(11/17: Richard Shaffer; Teresa Guerrero‑Urbano)

**Bath, Royal United Hospital**(23/53: Olivera Frim; Mark Beresford, Hugh Newman, Penny Kehagioglou)

**Belfast, Belfast City Hospital**(97/170: Joe O'Sullivan; Darren Mitchell, Poh Lin Shum, David Stewart, Suneil Jain)

**Birmingham, Birmingham Heartlands Hospital**(12/32: Anjali Zarkar)

**Birmingham, City Hospital**(11/24: Daniel Ford)

**Birmingham, Queen Elizabeth Hospital**(127/172: Nicholas James; Emilio Porfiri, Daniel Ford)

**Blackburn, Royal Blackburn Hospital**(32/61: Omi Parikh)

**Bolton, Royal Bolton Hospital**(8/24: Tony Elliott; Michael Pantelides)

**Boston, Pilgrim Hospital**(4/19: Thiagarajan Sreenivasan; Miguel Panades)

**Bournemouth, Royal Bournemouth Hospital**(52/84: Sue Brock; Joe Davies)

**Bradford, Bradford Royal Infirmary**(9/24: Simon Brown)

**Brighton, Royal Sussex County Hospital**(50/81: Angus Robinson; George Plataniotis, David Bloomfield, Marie Wilkins)

**Bristol, Bristol Haematology & Oncology Centre**(34/78: Amit Bahl; Mark Beresford, Paula Wilson, Serena Hilman, Chris Herbert)

**Burnley, Burnley General Hospital**(37/69: Natalie Charnley; Omi Parikh)

**Burton‑on‑Trent, Queens Hospital**(43/79: Shan Chetiyawardana; Dakshinamoorthy Muthukumar, Mike Smith‑Howell, Pugazhenthi Pattu, Prabir Chakraborti)

**Bury St Edmunds, West Suffolk Hospital**(7/17: Cathryn Woodward; Yvonne Rimmer)

**Cambridge, Addenbrooke's Hospital**(0/4: Danish Mazhar)

**Canterbury, Kent and Canterbury Hospital**(33/61: Carys Thomas; Natasha Mithal, Rakesh Raman, Albert Edwards)

**Cardiff, Velindre Hospital**(226/314: Malcolm Mason; Jim Barber, Jason Lester, John Staffurth, Jacob Tanguay, Nachiappan Palaniappan, Satish Kumar, Michael Button, Diana Mort)

**Carlisle, Cumberland Infirmary**(6/9: Anil Kumar; Norma Sidek)

**Chelmsford, Broomfield Hospital**(32/61: Abdel Hamid; Udaiveer Panwar, Priscilla Leone)

**Cheltenham, Cheltenham General Hospital**(2/17: Jo Bowen; Peter Jenkins)

**Chester, Countess of Chester Hospital**(27/66: Azman Ibrahim)

**Colchester, Essex County Hospital**(2/34: Bruce Sizer; Muthar Kumar)

**Coventry, University Hospital Coventry and Warwickshire**(0/30: Jane Worlding; Andrew Stockdale)

**Crewe, Leighton Hospital**(15/41: James Wylie)

**Darlington, Darlington Memorial Hospital**(17/34: Mohammed Kagzi; John Hardman, Clive Peedell, Tanmay Mukhopadhyay)

**Derby, Royal Derby Hospital**(52/100: Prabir Chakraborti; Dakshinamoorthy Muthukumar, Pugazhenthi Pattu)

**Doncaster, Doncaster Royal Infirmary**(5/22: Mymoona Alzouebi; Catherine Ferguson)

**Dorchester, Dorset County Hospital**(19/24: Perric Crellin; Stephen Andrews)

**Dudley, Russells Hall Hospital**(46/65: Pek Keng‑Koh; Prakash Ramachandra)

**Durham, University Hospital North Durham**(17/17: Rhona McMenemin)

**Eastbourne, Eastbourne District General Hospital**(32/52: Fiona McKinna)

**Edinburgh, Western General Hospital**(71/105: Duncan McLaren)

**Exeter, Royal Devon and Exeter Hospital**(102/152: Denise Sheehan; Rajaguru Srinivasan, Victoria Ford)

**Gillingham, Medway Maritime Hospital**(9/18: Henry Taylor)

**Glasgow, Beatson West of Scotland Cancer Centre**(223/291: Rob Jones; Martin Russell, Jan Wallace, John Graham, Rana Mahmood, Carolynn Lamb, Abdulla Al‑hasso, Balaji Venugopal)

**Guildford, Royal Surrey County Hospital**(71/106: Robert Laing; Julian Money‑Kyrle, Sara Khaksar, Katie Wood, Teresa Guerrero‑Urbano)

**Harlow, Princess Alexandra Hospital**(20/34: Nishi Gupta; Lucinda Melcher)

**Hereford, Hereford County Hospital**(35/53: Warren Grant; Audrey Cook)

**High Wycombe, Wycombe Hospital**(28/45: Ami Sabharwal; Andrew Protheroe, Philip Camilleri, Thinn Pwint, Gerard Andrade)

**Huddersfield, Huddersfield Royal Infirmary**(48/76: Uschi Hofmann)

**Hull, Castle Hill Hospital**(75/100: Matthew Simms; John Hetherington)

**Inverness, Raigmore Hospital**(43/72: Neil McPhail; Azmat Sadozye, Kay Kelly, Carol Macgregor)

**Ipswich, Ipswich Hospital**(42/84: Robert Brierly; Ramachandran Venkitaraman, Christopher Scrase, Gautam Banerjee)

**Keighley, Airedale General Hospital**(18/39: Simon Brown; Michael Crawford, Clara Sentamans)

**Kidderminster, Kidderminster General Hospital**(10/23: Mark Churn; Lisa Capaldi)

**Larbert, Forth Valley Royal Hospital**(3/22: Norma Sidek)

**Leeds, St James University Hospital**(**Leeds)**(16/54: William Cross; Stephen Prescott, David Bottomley, Alan Paul, Carmel Loughrey, Sunjay Jain, Ann Henry, Peter Whelan)

**Lincoln, Lincoln County Hospital**(6/32: Thiagarajan Sreenivasan; David Ballesteros‑Quintail, Miguel Panades, Karin Baria)

**Liverpool, Royal Liverpool University Hospital**(41/65: Zafar Malik; Chinnamani Eswar, Peter Robson)

**Liverpool, University Hospital Aintree**(7/16: Peter Robson)

**London, Charing Cross Hospital**(1/25: Alison Falconer)

**London, Guy's Hospital**(83/138: Simon Chowdhury; Peter Harper, Stephen Morris, Rick Popert, Ronald Beaney)

**London, Hammersmith Hospital**(3/4: Alison Falconer; Stephen Mangar)

**London, North Middlesex Hospital**(9/21: Jackie Newby; Anna Thompson, Farhad Neave, Stephen Karp)

**London, Queen Elizabeth Hospital**(12/18: Simon Hughes)

**London, Royal Free Hospital**(20/34: Maria Vilarino‑Varela; Katherine Pigott)

**London, Royal Marsden Hospital**(6/9: Vincent Khoo)

**London, St Bartholomews Hospital**(0/6: Karen Tipples; Paula Wells)

**London, St George's Hospital**(9/29: Lisa Pickering)

**London, St Mary's Hospital**(5/5: Alison Falconer; Simon Stewart)

**London, University College Hospital**(21/38: Ursula McGovern; Stephen Harland, Heather Payne)

**Maidstone, Maidstone Hospital**(54/86: Sharon Beesley; Amanda Clarke, Henry Taylor)

**Manchester, Christie Hospital**(96/142: Noel Clarke; Tony Elliott, James Wylie, Jacqueline Livsey, John Logue, Richard Cowan, Ananya Choudhurey)

**Manchester, Withington Hospital**(7/7: Vijay Sangar)

**Margate, Queen Elizabeth The Queen Mother Hospital**(4/15: Carys Thomas; Rakesh Raman, Natasha Mithal)

**Middlesbrough, James Cook University Hospital**(61/90: Clive Peedell; John Hardman, Hans Van der Voet, Devadasan Shakespeare, David Chadwick)

**Newcastle‑upon‑Tyne, Freeman Hospital**(0/56: Ashraf Azzabi; Rhona McMenemin, John Frew)

**Northwood, Mount Vernon Hospital**(58/98: Peter Hoskin; Roberto Alonzi, Peter Ostler, Nicola Anyamene, Robert Hughes, Jeanette Dickson, Charlotte Westbury)

**Nottingham, Nottingham University Hospitals, City Campus**(61/97: Santhanam Sundar; Jamie Mills, Eliot Chadwick)

**Nuneaton, George Eliot Hospital**(0/9: Andrew Chan)

**Oldham, Royal Oldham Hospital**(10/45: Jacqueline Livsey; Ananya Choudhury)

**Oxford, Churchill Hospital**(106/140: Andrew Protheroe; David J Cole)

**Poole, Poole Hospital**(22/52: Sue Brock; Joseph Davies, Joe Davies)

**Portsmouth, Queen Alexandra Hospital**(58/137: Joanna Gale)

**Preston, Royal Preston Hospital**(100/179: Alison Birtle; Omi Parikh, Marcus Wise)

**Reading, Royal Berkshire Hospital**(18/34: Paul Rogers; Helen O'Donnell, Richard B Brown)

**Redditch, Alexandra Hospital**(0/12: Joanna Hamilton)

**Romford, Queen's Hospital**(65/101: Stephanie Gibbs; Ramachandran Subramaniam)

**Salford, Salford Royal Hospital**(32/52: Noel Clarke; Maurice Lau, Tony Elliott, Anna Tran, Satish Maddineni)

**Scarborough, Scarborough General Hospital**(14/59: Mohan Hingorani)

**Sheffield, Weston Park Hospital**(68/99: Catherine Ferguson; Peter Kirkbride, Mymoona Alzouebi, Tathagata Das)

**Shrewsbury, Royal Shrewsbury Hospital**(76/132: Narayanan Srihari; Ravi Prashant)

**South Shields, South Tyneside District Hospital**(2/4: Ashraf Azzabi)

**Southampton, Southampton General Hospital**(32/66: Catherine Heath; Simon Crabb, Matthew Wheater)

**Southend, Southend University Hospital**(76/102: David Tsang; Imtiaz Ahmed, Olivia Chan, Naveed Sarwar)

**Southport, Southport and Formby District General Hospital**(15/37: Neeraj Bhalla; Chinnamani Eswar, Asha Sivapalasuntharam)

**St Leonards‑on‑Sea, Conquest Hospital**(25/31: Fiona McKinna; Kathryn Lees, Sharon Beesley)

**Stevenage, Lister Hospital**(19/27: Robert Hughes)

**Stockport, Stepping Hill Hospital**(58/88: John Logue; Adebanji Adeyoju)

**Stockton‑on‑Tees, University Hospital of North Tees**(2/10: Devadasan Shakespeare)

**Stoke‑on‑Trent, Royal Stoke Hospital**(22/57: Fawzi Adab; Rajanee Bhana)

**Sunderland, Sunderland Royal Hospital**(29/34: Ashraf Azzabi; Ian Pedley)

**Sutton Coldfield, Good Hope Hospital**(3/14: Daniel Ford)

**Sutton, Royal Marsden Hospital**(73/126: David Dearnaley; Chris Parker, Robert Huddart, Vincent Khoo)

**Sutton‑in‑Ashfield, King's Mill Hospital**(23/36: Daniel Saunders; Georgina Walker)

**Swansea, Singleton Hospital**(115/160: John Wagstaff; Gianfilippo Bertelli, Delia Pudney, Mau‑Don Phan)

**Swindon, Great Western Hospital**(21/41: Omar Khan; David J Cole, Esme Hill)

**Taunton, Musgrove Park Hospital**(65/103: Emma Gray; John Graham, Mohini Varughese, Manjusha Keni, George Plataniotis)

**Torquay, Torbay District General Hospital**(68/114: Anna Lydon; Rajaguru Srinivasan)

**Warrington, Warrington Hospital**(54/93: Isabel Syndikus; Shaun Tolan)

**Warwick, Warwick Hospital**(0/13: Andrew Stockdale)

**Weston Super Mare, Weston General Hospital**(6/12: Serena Hilman)

**Whitehaven, West Cumberland Hospital**(1/1: Anil Kumar; Jonathon Nicoll)

**Wigan, Royal Albert Edward Infirmary**(13/25: Anna Tran; Richard Cowan)

**Wirral, Clatterbridge Centre for Oncology**(57/107: Shaun Tolan; John Littler, Isabel Syndikus, Amir Montazeri, Azman Ibrahim)

**Wolverhampton, New Cross Hospital**(0/19: Ian Sayers)

**Worcester, Worcestershire Royal Hospital**(24/44: Lisa Capaldi; Jo Bowen)

**Worthing, Worthing Hospital**(51/75: Ashok Nikapota; David Bloomfield, Fiona Castell)

**Yeovil, Yeovil District Hospital**(0/4: Geoffrey Sparrow; Emma Gray)

**SWITZERLAND**

**Aarau, Hirslanden Medical Centre**(3/3: Razvan Popescu)

**Basel, Universitätsspital Basel**(1/3: Cyrill Rentsch; Bettina Seifest)

**Berne, Inselspital**(**University Hospital Berne)**(1/3: George Thalmann; Beat Roth)

**Chur, Kantonsspital Graubünden**(17/24: Raeto Strebel; Richard Cathomas)

**Lausanne, Centre Hospitalier Universitaire Vaudois**(**CHUV)**(3/5: Dominik Berthold; Patrice Jichlinski, Fernanda Herrera)

**St Gallen, Kantonsspital St Gallen**(5/8: Daniel Engeler; Stefan Prensser)

**Zurich, Triemlispital**(1/1: Donat Durr; Daniele Siciliano)

**Independent Data Monitoring Committee:** John Yarnold (from 2014, chair), Chris Williams (to 2014, chair), Doug Altman, Reg Hall Bertrand Tombal

**Trial Steering Committee:** Jonathan Ledermann, Jim Paul, David Kirk (to 2015), John Fitzpatrick (2014)

**MRC Clinical Trials Unit at UCL: *Statisticians*** – Matthew Sydes, Max Parmar, Melissa Spears, Christopher Brawley, Gordana Jovic, Rachel Jinks, Patrick Royston, Sophie Barthel, Babak Choodari-Oskooei, Daniel Bratton; ***Trial Management*** – Claire Amos, Nafisah Atako; Sharon Naylor, Neil Kelk, James Latham, Jacqui Nuttall, Karen Sanders, Tom Fairfield, Charlene Griffiths, Francesca Schiavone, Alanna Brown, Orla Prendiville, Katie Ward, Anna Herasimtschuk, Claire Murphy, Chris Wanstall, Arlen Wilcox, Charlotte Tyson, Michelle Buckner, Mazna Anjum, Joanna Calvert; ***Data Management*** –Neil Kelk, Karen Sanders, Tom Fairfield, Charlene Green, Emma Donoghue, Tim Smith, Jacque Millett, Shama Hassan, Philip Pollock, Richard Gracie, Laura Van Dyck, Charlene Green, Elizabeth Clark, Sara Peres, Hannah Gardner, Dominic Hague, Katie Ward, Peter Vaughan, Andrew Whitney, Eva Ades, Hannah Babiker, Carly Au, Nargis Begum, Jenna Grabey, Danielle Johnson, Amy Fiddament, Stephanie Wetton, Zhorah Khan; ***Data Services*** – Nadine Van Looy, Zaheer Islam, Sajad Khan; ***Others*** - Sarah Meredith, Ruth Langley

**Novartis:** Support for the STAMPEDE trial has been provided by Novartis Pharmaceuticals UK Limited.

**Sanofi-Aventis:** Christine Geffriaud-Ricouard, Paul Cadle. Support for the STAMPEDE study has been provided by Sanofi-Aventis.

**Cancer Research UK:** Kate Law

**ACKNOWLEDGEMENTS**

DD, CP, GA and JdeB acknowledge support from the NIHR to the Royal Marsden NHS Trust and The Institute of Cancer Research Biomedical Research Centre

## References

1. James N, Spears M, Clarke N, et al. Survival with Newly Diagnosed Metastatic Prostate Cancer in the “Docetaxel Era”: Data from 917 Patients in the Control Arm of the STAMPEDE Trial (MRC PR08, CRUK/06/019). *European Urology.* 2015;67(6):1028-1038.

2. James N, Spears M, Clarke N, et al. Failure-Free Survival and Radiotherapy in Patients With Newly Diagnosed Nonmetastatic Prostate Cancer. Data From Patients in the Control Arm of the STAMPEDE Trial. *JAMA Oncol.* 2016;2(3):348-357.

3. James N, Sydes M, Clarke N, et al. Addition of docetaxel, zoledronic acid, or both to first-line long-term hormone therapy in prostate cancer (STAMPEDE): survival results from an adaptive, multiarm, multistage, platform randomised controlled trial. *Lancet.* 2016;387:1163–1177.

4. Afshar M, Al-Alloosh F, Pirrie S, Rowan C, James N, Porfiri E. Predictive factors for response to abiraterone in metastatic castration refractory prostate cancer. *Anticancer Research.* 2015;35(2):1057-1063.

5. Papaioannou D, Rafia R, Rathbone J, et al. Rituximab for the first-line treatment of stage III–IV follicular lymphoma (review of Technology Appraisal No. 110): a systematic review and economic evaluation. *Health Technology Assessment.* 2012;16(37).

6. Office of National Statistics. *2014-based Mortality rates, UK, calendar year.* 2016.

7. Hanks G, Pajak T, Porter A, et al. Phase III Trial of Long-Term Adjuvant Androgen Deprivation After Neoadjuvant Hormonal Cytoreduction and Radiotherapy in Locally Advanced Carcinoma of the Prostate: The Radiation Therapy Oncology Group Protocol 92– 02. *Journal of Clinical Oncology.* 2003;21.

8. Lawton C, Dignam J, Hanks G, et al. Duration of Androgen Deprivation in Locally Advanced Prostate Cancer: Long-term Update of NRG Oncology/RTOG 9202 (abstract only). *International Journal of Radiation Oncology, Biology, Physics.* 2015;93(3):s44.

9. Widmark A, Klepp O, Solberg A, et al. Endocrine treatment, with or without radiotherapy, in locally advanced prostate cancer (SPCG-7/SFUO-3): an open randomised phase III trial. *Lancet.* 2009;373:301-308.

10. Nelson JB, Love W, Chin JL, et al. Phase 3, randomized, controlled trial of atrasentan in patients with nonmetastatic, hormone‐refractory prostate cancer. *Cancer.* 2008;113(9):2478-2487.

11. Hussain M, Tangen C, Berry D, et al. Intermittent versus Continuous Androgen Deprivation in Prostate Cancer. *The New England Journal Of Medicine.* 2013;368(14).

12. Ryan C, Smith M, Fizazi K, et al. Abiraterone acetate plus prednisone versus placebo plus prednisone in chemotherapy-naive men with metastatic castration-resistant prostate cancer (COU-AA-302): fi nal overall survival analysis of a randomised, double-blind, placebo-controlled phase 3 study. *Lancet Oncology.* 2015;16:152-160.

13. Dolan P. Modeling Valuations for EuroQol Health States. *Medical Care.* 1997;35(11):1095-1108.

14. Kind P, Dolan P, Gudex C, Williams A. Variations in population health status: results from a United Kingdom national questionnaire survey. *BMJ.* 1998;316:736–741.

15. van Buuren S, Groothuis-Oudshoorn K. mice: Multivariate Imputation by Chained Equations in R. *Journal of Statistical Software.* 2011;45(3).

16. Rintoul R, Ritchie A, Edwards J, et al. Efficacy and cost of video-assisted thoracoscopic partial pleurectomy versus talc pleurodesis in patients with malignant pleural mesothelioma (MesoVATS): an open-label, randomised, controlled trial. *Lancet.* 2014;384:1118-1127.

17. Basu A, Manca A. Regression estimators for generic health-related quality of life and quality-adjusted life years. *Med Decis Making.* 2012;21(1):56-69.

18. Drugs and pharmaceutical electronic market information (eMit). [*https://wwwgovuk/government/publications/drugs-and-pharmaceutical-electronic-market-information-emit*](https://wwwgovuk/government/publications/drugs-and-pharmaceutical-electronic-market-information-emit)*.*

19. British National Formulary (BNF). [*https://wwwevidencenhsuk/formulary/bnf/current*](https://wwwevidencenhsuk/formulary/bnf/current)*.*

20. Lord J, Willis S, Eatock J, et al. Economic modelling of diagnostic and treatment pathways in National Institute for Health and Care Excellence clinical guidelines: the Modelling Algorithm Pathways in Guidelines (MAPGuide) project. *Health Technology Assessment.* 2013;17.58.

21. Cabazitaxel for hormone-relapsed metastatic prostate cancer treated with docetaxel (TA391). *National Institute for Health and Care Excellence.* 2016.

22. NHS reference costs 2014 to 2015. *Department of Health.* 2015;<https://www.gov.uk/government/publications/nhs-reference-costs-2014-to-2015>.

23. Abiraterone for treating metastatic hormone-relapsed prostate cancer before chemotherapy is indicated (TA387). *National Institute for Health and Care Excellence.* 2016.

24. Radium-223 dichloride for treating hormone-relapsed prostate cancer with bone metastases (TA376). *National Institute for Health and Care Excellence.* 2016.

25. James N, Pirrie S, Pope A, et al. TRAPEZE: a randomised controlled trial of the clinical effectiveness and cost-effectiveness of chemotherapy with zoledronic acid, strontium-89, or both, in men with bony metastatic castration-refractory prostate cancer. *Health Technol Assess.* 2015;15.

26. Tannock I, deWit R, Berry W, et al. Docetaxel plus Prednisone or Mitoxantrone plus Prednisone for Advanced Prostate Cancer. *The New England Journal of Medicine.* 2004;351:1502-1512.

27. deBono J, Logothetis C, Molina A, et al. Abiraterone and Increased Survival in Metastatic Prostate Cancer. *The New England Journal of Medicine* 2011;364:1995-2005.

28. Beer T, Armstrong A, Rathkopf D, et al. Enzalutamide in Metastatic Prostate Cancer before Chemotherapy. *The New England Journal of Medicine.* 2014;371:424-433.

29. Scher H, Fizazi K, Saad F, et al. Increased Survival with Enzalutamide in Prostate Cancer after Chemotherapy. *The New England Journal of Medicine* 2012;367:1187-1197.

30. deBono J, Oudard S, Ozguroglu M, et al. Prednisone plus cabazitaxel or mitoxantrone for metastatic castration-resistant prostate cancer progressing after docetaxel treatment: a randomised open-label trial *Lancet.* 2010;376:1147-1154.

31. Curtis L, Burns A. Unit Costs of Health and Social Care. *Personal Social Services Research Unit (PSSRU), University of Kent, Canterbury.* 2015.

32. Enzalutamide for treating metastatic hormone-relapsed prostate cancer before chemotherapy is indicated (TA377). *National Institute for Health and Care Excellence.* 2016.

33. Ramsay C, Adewuyi T, Gray J, et al. Ablative therapy for people with localised prostate cancer: a systematic review and economic evaluation. *Health Technology Assessment.* 2015;19(49).

34. Hounsome L, Gillatt D, Persad R, Verne J. Hospital care for cancer patients in the last year of life. *South West Public Health Observatory.* 2012.

35. Round J, Jones L, Morris S. Estimating the cost of caring for people with cancer at the end of life: A modelling study. *Palliative Medicine.* 2015;29(10).

36. Gillessen S, Attard G, Beer TM, et al. Management of patients with advanced prostate cancer: the report of the Advanced Prostate Cancer Consensus Conference APCCC 2017. *European Urology.* 2017.

37. Davis S, Stevenson M, Tappenden P, Wailoo A. NICE DSU Technical Support Document 15: Cost-Effectiveness Modelling Using Patient-Level Simulation. *NICE Decision Support Unit.* 2014;ScHARR, University of Sheffield.

38. Vale C, Burdett S, Rydzewska L, et al. Addition of docetaxel or bisphosphonates to standard of care in men with localised or metastatic, hormone-sensitive prostate cancer: a systematic review and meta-analyses of aggregate data. *Lancet Oncology.* 2016;17(2):243-256.
